# Blocking interleukin-1 receptor type 1 (IL-1R1) signaling in hepatocytes slows down diethylnitrosamine-induced liver tumor growth in obese mice

**DOI:** 10.1097/HC9.0000000000000568

**Published:** 2024-11-29

**Authors:** Nadine Gehrke, Lea J. Hofmann, Beate K. Straub, Dirk A. Ridder, Ari Waisman, Leonard Kaps, Peter R. Galle, Jörn M. Schattenberg

**Affiliations:** 1 I. Department of Medicine, University Medical Center of the Johannes Gutenberg, University Mainz, Mainz, Germany; 2 Institute of Pathology, University Medical Center of the Johannes Gutenberg, University Mainz, Mainz, Germany; 3 Institute for Molecular Medicine, University Medical Center of the Johannes Gutenberg, University Mainz, Mainz, Germany; 4 Research Center for Immunotherapy, University Medical Center of the Johannes Gutenberg University Mainz, Mainz, Germany; 5 Department of Internal Medicine II, Saarland University Medical, Homburg, Germany; 6 Department of Dermatology, University Medical Center of the Johannes Gutenberg University Mainz, Mainz, Germany; 7 Saarland University, Saarbrücken, Germany

**Keywords:** hepatocarcinogenesis, insulin resistance, interleukin-1 receptor type 1 (IL-1R1), metabolic inflammation, metabolic dysfunction–associated steatohepatitis (MASH)

## Abstract

**Background:**

An increasing number of HCC develops in the context of metabolic dysfunction-associated steatotic liver disease and its inflammatory form, metabolic dysfunction–associated steatohepatitis, even in the absence of cirrhosis. Chronic metabolic inflammation is the driving force of metabolic dysfunction–associated steatotic liver disease progression and a key factor in hepatocarcinogenesis. Given the prominent role of IL-1 signaling in inflammation and metabolic diseases, we investigated the relevance of the hepatocyte-specific IL-1 receptor type 1 knockout in metabolic dysfunction–associated steatohepatitis–related noncirrhotic HCC.

**Methods:**

For HCC induction, *Il1r1* ^Hep−/−^ mice received a single i.p. injection of diethylnitrosamine at 2 weeks and were fed with high-fat plus high-carbohydrate diet, starting from 4 weeks. After 18 weeks of diet intervention, mice were sacrificed, and macroscopic and microscopic tumor loads were assessed.

**Results:**

Knockout of the hepatic IL-1 receptor type 1 pathway significantly reduced liver tumor growth. *Il1r1* ^Hep−/−^ mice were also less susceptible to hepatic steatosis, insulin resistance, and associated hepatic c-Jun N-terminal kinase activation than their wild-type (WT) littermates. Reduced Ki-67 and cyclin D1 levels, as well as decreased phosphorylation of signal transducer and activator of transcription 3, occur in *Il1r1* ^Hep−/−^ livers, lowering cancer cell proliferation and growth. Additionally, in *Il1r1* ^Hep−/−^ livers, the chemokine (C-X-C motif) ligand 1/2-driven accumulation of myeloid-derived suppressor cells and CD8^+^ T-cell infiltration were reduced compared to the wild type.

**Conclusions:**

Metabolic inflammation mediated by the hepatocytic IL-1 receptor type 1 is a cofactor in mutagenic hepatocarcinogenesis. Targeting IL-1 signaling could be an adjunct strategy to the current immunomodulatory HCC treatments.

## INTRODUCTION

HCC is the sixth most common cancer and third leading cause of cancer-related mortality worldwide.^1^ The incidence of HCC linked to metabolic dysfunction–associated steatotic liver disease (MASLD) and metabolic dysfunction–associated steatohepatitis (MASH) is expected to rise significantly.^2^ Hepatocarcinogenesis can be driven by inflammation even in the absence of cirrhosis. However, the molecular mechanisms underlying MASLD/MASH-associated HCC remain to be elucidated. Experimental animal studies suggest that excessive lipid accumulation in MASLD, coupled with insulin resistance, may promote liver cancer by inducing chronic, low-grade metabolic inflammation. This process involves the release of multiple proinflammatory cytokines, vasoactive factors, and pro-oxidant molecules, consequently triggering chronic exposure to oncogenic signals that promote the development of HCC.^3^

The proinflammatory cytokines IL-1α and IL-1β, which are produced by both parenchymal and nonparenchymal liver cells, especially KCs, under inflammatory and/or stress conditions, have been identified as important mediators in metabolically driven liver inflammation and control of lipid and glucose metabolism.^4,5,6,7,8^ Similarly, IL-1 released from adipose tissue has been suggested to adversely affect the liver.^9,10^ Using hepatocyte-specific interleukin-1 receptor type 1 (IL-1R1)-deficient (*Il1r1* ^Hep−/−^) mice, we recently generated evidence that blocking the IL-1 pathway selectively in hepatocytes protects against metabolic liver injury and improves insulin sensitivity and adipose tissue inflammation in obese mice.^11^ Despite the relevance of IL-1R1 signaling in HCC, its role in MASLD-driven hepatocarcinogenesis has not been addressed before.

IL-1α/β signaling via cell surface IL-1R1 activates NF-κB, c- JNK, extracellular signal-regulated kinases (ERKs), and p38 mitogen-activated protein kinase (MAPK) signaling pathways, which regulate multiple cellular processes, including inflammation, proliferation, angiogenesis, and tissue repair. Thus, hepatic activation of IL-1R1 due to nonresolving metabolic inflammation in MASLD may favor tumor development. Several studies have indicated the contribution of IL-1 signaling to malignant transformation in chronic liver disease.^12,13,14,15^ Genetic polymorphisms at the IL-1β locus in humans have been shown to be strongly associated with susceptibility to HCC.^16^ Similarly, upregulation of IL-1R1 expression in human HCC tissues was found to correlate with tumor size and higher TNM stage.^17^ Thus, IL-1/IL-1R1 blockade could be an adjunctive strategy for immunomodulation in HCC.

In this study, we investigated the effect of hepatocyte-specific IL-1R1 knockout on metabolic inflammation–driven hepatocarcinogenesis using a transgenic mouse model. This model combined the chemotoxic agent diethylnitrosamine (DEN) with a high-fat, high-carbohydrate diet (HFD). We hypothesized that the knockout might alleviate MASLD-related metabolic disturbances and reduce tumor burden.

## METHODS

### Animal model including ethical approval statement

All animals were housed and bred at the animal facility of the University Medical Center Mainz according to the criteria outlined in the “Guide for the Care and Use of Laboratory Animals.” Studies, including selected sample sizes, were approved by the Committee for Experimental Animal Research (Landesuntersuchungsamt Rheinland-Pfalz, Koblenz, Germany, approval ID: G-18-1-066). HCC induction in *Il1r1* ^Hep−/−^ mice^18^ and wild-type (WT) littermates and analysis of hepatic tissue, including macroscopic and microscopic tumor assessment, are detailed in Supplemental Materials and Methods, http://links.lww.com/HC9/B79. Composition of the experimental diets is listed in Supplemental Table S1, http://links.lww.com/HC9/B79.

### Serological analysis

Serum was obtained from 16- to 18-hour fasted mice by cardiac puncture and assayed for levels of ALT, AST, lactate dehydrogenase (LDH), total cholesterol, triglycerides, and glucose using a standard analyzer (Hitachi 917, Roche, Basel, Switzerland). ELISA kits were used to measure insulin (MilliporeSigma, St. Louis, MI), nonester fatty acids, and alpha-fetoprotein (AFP, MyBioSource, San Diego, CA) in the sera. The homeostasis model assessment of insulin resistance and adipose tissue insulin resistance indices are detailed in Supplemental Materials and Methods, http://links.lww.com/HC9/B79. The measurement of cytokines and chemokines, including IL-1α/β, IL-6, monocyte chemotactic protein-1 (MCP-1/CCL2), and C-X-C motif ligand 1 (CXCL1), was performed using bead-based multiplex immunoassays (BD Biosciences, Heidelberg, Germany), as described.^19^

### Quantitative real-time-PCR

Isolation of total RNA, cDNA synthesis, and quantitative real-time-PCR were performed as described.^20^ All samples were analyzed in duplicate. Roche LightCycler software (LightCycler 480 Software Release 1.5.0, Roche) was used to perform advanced relative quantification analysis using the 2^(-ΔΔC(T))^ method. Expression data were normalized to the housekeeping gene *Gapdh* encoding glyceraldehyde-3-phosphate dehydrogenase (mouse primer sequences from Qiagen, Hilden, Germany), which was stably expressed and calculated as fold change overexpression in PBS+control diet (CD)-treated WT mice, which was considered 1. Target genes and primer sequences used for RT-qPCR (Eurofins Genomics, Ebersberg, Germany) are listed in Supplemental Table S2, http://links.lww.com/HC9/B79.

### Immunoblotting

Proteins were isolated and separated as described.^21^ The primary antibodies used were p38 MAPK, phosho-p38 MAPK (Thr180/Tyr182), p44/42 MAPK (ERK1/2), phospho-p44/42 MAPK (ERK1/2) (Thr202/Tyr204), stress-activated kinases/JNK, phospho- stress-activated kinases/JNK (Thr183/Tyr185), phospho-STAT3 (Tyr705) (all obtained from Cell Signaling Technology, Danvers, MA, Cat #9212, 4511, 9102, 9101, 9252, 4668, 9131), and α-tubulin (Abcam, Cambridge, UK, Cat #ab4074). Membranes were incubated with anti-rabbit secondary antibody conjugated to horseradish peroxidase (Santa Cruz Biotechnology, Cat #sc-2054). Clarity Western ECL Substrate (Bio-Rad, Feldkirchen, Germany) or WesternBright Chemilumineszenz Substrate Quantum (Biozym, Hess. Oldendorf, Germany) was used for visualization. Adobe Acrobat Professional software program (Adobe Systems Incorporated, San Jose, CA) was used to cut the immunoblot images to the desired size. No postprocessing of the images was performed. Densitometric analysis was performed using the ImageJ software (National Institutes of Health).

### Determination of the hepatic malondialdehyde content, caspase 3, and NF-κB p65 activities

Malondialdehyde (MDA) levels in the whole liver tissue were detected and quantitated using a Lipid Peroxidation (MDA) Colorimetric Assay Kit (BioVision, Milpitas, CA) according to the manufacturer’s protocol. Caspase 3 activity was determined by chromogenic peptide substrate cleavage (Ac-DEVD-AFC; Biomol, Hamburg, Germany), as described.^19,22^ NF-κB p65 activity was measured in duplicate using a TransAM NF-κB Family Kit (Active Motif, Carlsbad, CA).

### Flow cytometric analysis

Isolation of intrahepatic immune cells and flow cytometric analysis were performed as described (all antibodies were obtained from BioLegend, San Diego, CA).^21^

### Isolation of primary hepatocytes and ex vivo stimulation

Hepatocytes were isolated and cultured as described.^20^ After 24 hours, cells were treated with DEN (0–10 mM) in the absence or presence of recombinant mouse (rm) IL-1α or IL-1β protein (10 ng/mL, both from R&D Systems, Minneapolis, MN). The pan-caspase inhibitor zVAD (50 µM; Enzo Life Sciences, Lörrach, Germany) was added 1 hour before DEN treatment. Cell survival was assessed using the MTT assay (Sigma-Aldrich) after 24 hours. Untreated hepatocytes were used as baseline controls.

### Statistical analysis

Statistical details are summarized in the Supplemental Materials and Methods, http://links.lww.com/HC9/B79.

## RESULTS

### Inhibition of hepatocytic IL-1R1 restores insulin sensitivity in obese mice

**FIGURE 1:**
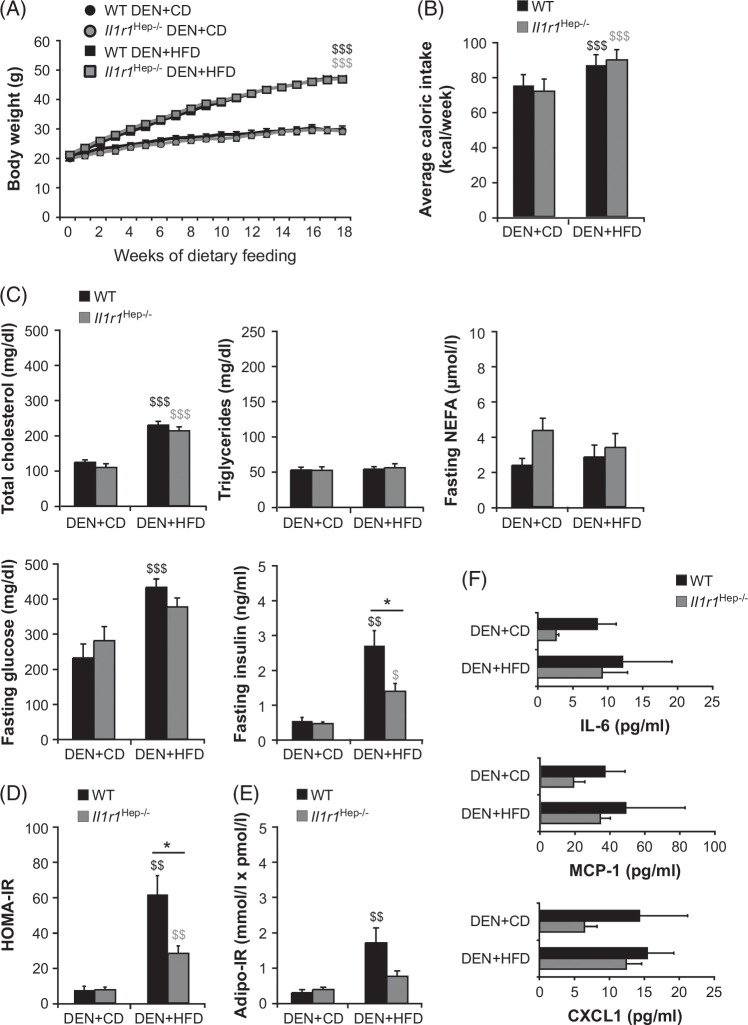
Body weight gain and systemic metabolic alterations in DEN-injected *Il1r1* ^Hep−/−^ and WT mice fed the HFD for 18 weeks. DEN was given intraperitoneally to 2-week-old male *Il1r1* ^Hep−/−^ mice and WT littermates. From 6 weeks of age, the mice were fed either a HFD or a corresponding CD. (A) Body weight curve and (B) mean caloric intake during the feeding period. (C) At week 18 of feeding, sera from overnight-fasted *Il1r1* ^Hep−/−^ and WT mice were analyzed for levels of total cholesterol, triglycerides, NEFA, glucose, and insulin. (D) HOMA-IR and (E) Adipose tissue insulin resistance indices were determined. (F) The concentrations of IL-6, MCP-1, and CXCL1 were measured in the sera by bead-based multiplex immunoassays. Data in A–D and F represent mean±SEM of n=7 WT DEN+CD, n=16 WT DEN+HFD, n=7 *Il1r1* ^Hep−/−^ DEN+CD, n=16 *Il1r1* ^Hep−/−^ DEN+HFD mice at 24 weeks of age. Data in C (NEFA) and E represent mean±SEM of n=7 WT DEN+CD, n=14 WT DEN+HFD, n=7 *Il1r1* ^Hep−/−^ DEN+CD, n=10 *Il1r1* ^Hep−/−^ DEN+HFD mice at 24 weeks of age. *p<0.05 for WT versus *Il1r1* ^Hep−/−^, and ^$^ *p*<0.05, ^$$^ *p*<0.01, ^$$$^ *p*<0.001 for DEN+CD versus DEN+HFD using two-way method of ANOVA followed by Bonferroni multiple comparisons tests (A–C, E, and F) or Kruskal-Wallis H test followed by pairwise Bonferroni-corrected Mann-Whitney *U* tests (C [insulin] and D). Abbreviations: CD, control diet; CXCL, C-X-C motif ligand; DEN, diethylnitrosamine; HFD, high-carbohydrate diet; HOMA-IR, homeostasis model assessment of insulin resistance; MCP-1, monocyte chemotactic protein-1; NEFA, non-ester fatty acid; WT, wild type.

The body weight curves and average food consumption were almost identical for DEN-injected *Il1r1* ^Hep−/−^ and WT mice fed each diet over the 18-week feeding period (Figure 1A, B). Owing to the significantly higher caloric intake from the HFD, ad-libitum access to the HFD induced significant weight gain in both genotypes compared with the CD, with no difference between the genotypes (Figure 1A, B). Concomitantly with the obese phenotype, HFD-fed *Il1r1* ^Hep−/−^ and WT mice developed hypercholesterolemia and hyperglycemia (Figure 1C). *Il1r1* ^Hep−/−^ mice showed lower levels of fasting insulin, homeostasis model assessment of insulin resistance (Figure 1D), and adipose tissue insulin resistance indices (Figure 1E) following HFD feeding, albeit with comparable levels of circulating nonester fatty acid (Figure 1C). Thus, despite HFD feeding, *Il1r1* ^Hep−/−^ mice remained insulin-sensitive, as previously shown.^11^

Serum levels of IL-6, MCP-1, and CXCL1 did not differ between the DEN+HFD and DEN+HFD mice, regardless of their genotype (Figure 1F). However, there was a trend for higher levels of inflammatory markers in WT mice, which may suggest increased attraction of immune cells to the liver and other potentially metabolically challenged peripheral tissues. In contrast, neither IL-1α nor IL-1β was detected in mouse sera (data not shown).

### Blocking hepatic IL-1R1 reduces hepatic steatosis and slows DEN-initiated liver tumor growth in obese mice

**FIGURE 2:**
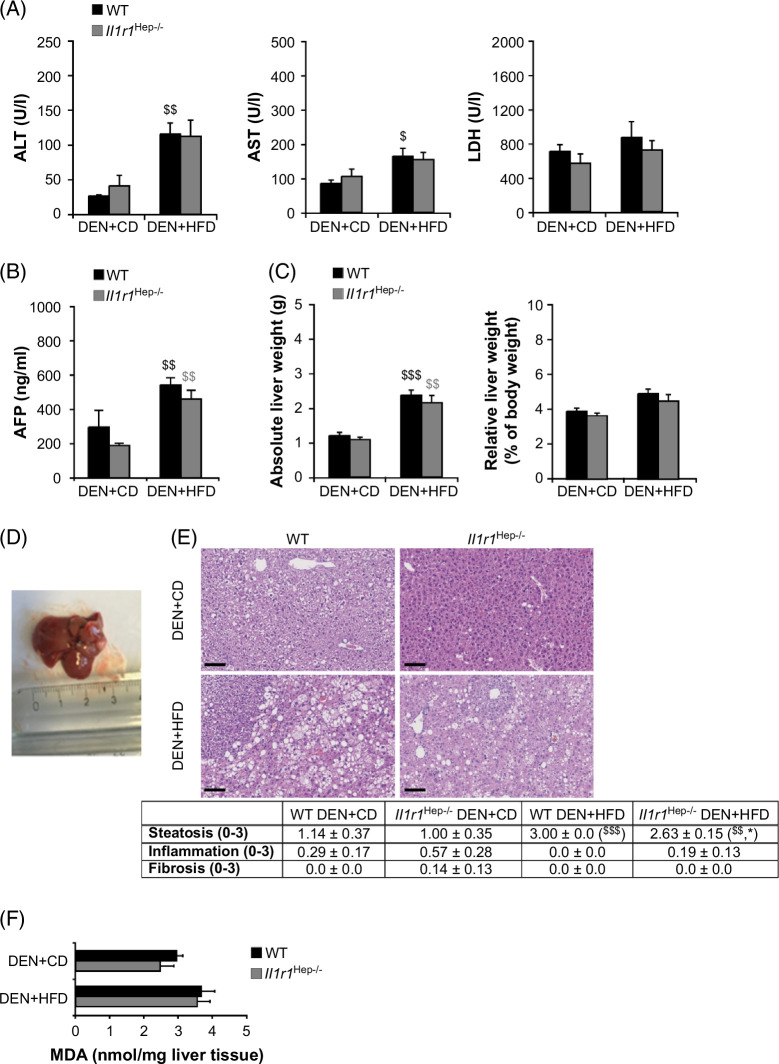
Serum liver enzymes, AFP levels and MASLD grade in DEN-injected *Il1r1* ^Hep−/−^ and WT mice at week 18 of HFD feeding. (A) Serum levels of ALT, AST, LDH, and (B) AFP, (C) absolute liver weight, liver-to-body weight ratio, (D) representative macroscopic liver appearance, (E) representative liver histology by H&E staining (magnification: ×10, scale bar: 100 µm) and pathological scores, and (F) MDA levels in tumors surrounding liver tissue homogenates from 24-week-old *Il1r1* ^Hep−/−^ and WT mice, which had received DEN+HFD or DEN+CD. (Data in A–C and E represent mean±SEM of n=7 WT DEN+CD, n=16 WT DEN+HFD, n=7 *Il1r1* ^Hep−/−^ DEN+CD, n=16 *Il1r1* ^Hep−/−^ DEN+HFD mice at 24 weeks of age. Data in F represent mean±SEM of n=7 WT DEN+CD, n=16 WT DEN+HFD, n=7 *Il1r1* ^Hep−/−^ DEN+CD, n=14 *Il1r1* ^Hep−/−^ DEN+HFD mice at 24 weeks of age. *p<0.05 for WT versus *Il1r1* ^Hep−/−^, and ^$^ *p*<0.05, ^$$^ *p*<0.01, ^$$$^ *p*<0.001 for DEN+CD versus DEN+HFD using Kruskal-Wallis H test followed by pairwise Bonferroni-corrected Mann-Whitney *U* tests (A and E) or two-way method of ANOVA followed by Bonferroni multiple comparisons tests (B, C, and F).) Abbreviations: AFP, alpha-fetoprotein; CD, control diet; DEN, diethylnitrosamine; HFD, high-carbohydrate diet; LDH, lactate dehydrogenase; WT, wild type.

HFD intake aggravated hepatic injury from DEN in both genotypes, as evidenced by higher serum ALT, AST, and LDH levels than their CD-fed counterparts (Figure 2A). Additionally, while serum AFP was barely detectable in age-matched *Il1r1* ^Hep−/−^ and WT PBS+CD controls, all DEN-treated mice showed significantly higher AFP levels (*p*<0.01) in the DEN+HFD versus DEN+CD groups (Figure 2B). The increase in AFP was most pronounced in WT DEN+HFD mice, but the difference between WT and *Il1r1* ^Hep−/−^ mice did not reach statistical significance.

The livers were excised, weighed, and analyzed to further assess macroscopic liver appearance and pathological changes. All DEN-injected mice developed hepatomegaly when fed an HFD, regardless of the genotype (Figure 2C). This was due to massive fat accumulation, as evidenced by marked discoloration of the liver tissue and numerous macroscopically visible tumor nodules on the liver surface (Figure 2D). Histological analysis of hematoxylin and eosin–stained liver sections validated mixed macrovesicular and microvesicular hepatic steatosis from the HFD, which was moderate to severe in both genotypes after 18 weeks on the HFD but significantly less pronounced in *Il1r1* ^Hep−/−^ mice (Figure 2E). Importantly, no necroinflammation or fibrosis, suggestive of inflammatory MASH, was observed histologically in *Il1r1* ^Hep−/−^ or WT livers (Figure 2E). Similarly, consumption of HFD led to only slightly elevated levels of liver MDA, irrespective of IL-1R1 (Figure 2F), indicating mild chronic metabolic stress from HFD feeding.^23^ Thus, hepatocyte IL-1R1 acts as an initiating factor for hepatosteatosis in obese mice, as previously observed in vivo and in vitro.^11^

quantitative real-time-PCR analysis revealed that IL-1R1 deficiency had no major influence on the expression of genes encoding primary regulators of de novo lipogenesis, such as peroxisome proliferator-activated receptor-γ and sterol regulatory element-binding transcription factor1c (Supplemental Table S3, http://links.lww.com/HC9/B79). However, IL-1R1 knockout was associated with higher hepatic peroxisome proliferator-activated receptor-α, carnitine palmitoyltransferase 1, and farnesoid X receptor-α levels, irrespective of dietary intervention, in parallel with increased heme oxygenase-1 (HO-1) induction from DEN and HFD (Supplemental Table S3, http://links.lww.com/HC9/B79). This agrees with our previous report that verified improved mitochondrial functionality and enhanced lipid catabolic processes in *Il1r1* ^Hep−/−^ livers.^11^

**FIGURE 3:**
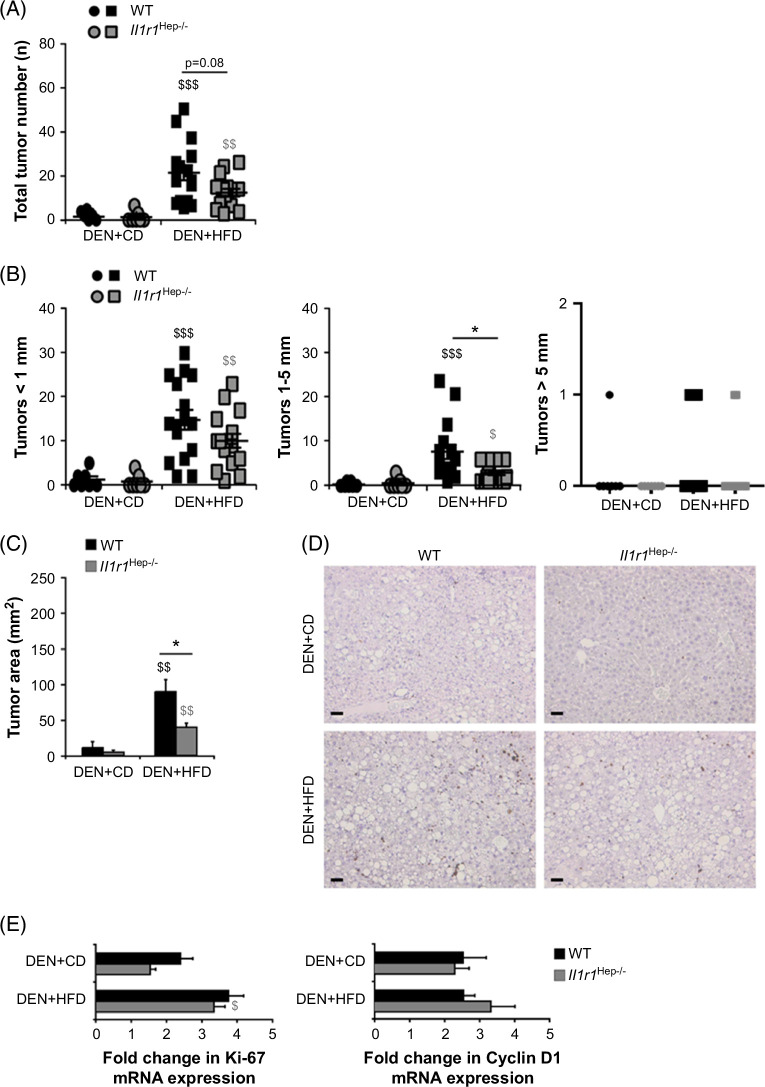
Effect of HFD on liver tumorigenesis and cell proliferation in DEN-injected *Il1r1* ^Hep−/−^ and WT mice. Livers from 24-week-old *Il1r1* ^Hep−/−^ and WT mice, which had received DEN+HFD or DEN+CD, were evaluated for tumors. (A) Nodules visible at the surface upon macroscopic examination, (B) diameter of the visible nodules, and (C) calculated total tumor area. (D) Representative histological images of Ki-67 immune histochemical stainings (magnification: ×10, scale bar: 5000 µm). (E) Gene expression analysis of Ki-67 and cyclin D1 in tumors surrounding liver tissue lysates. Expression data were normalized to the housekeeping gene *Gapdh*, which was stably expressed, and calculated as fold change overexpression in age-matched WT PBS+CD mice, which was considered 1. Data in A–C represent mean±SEM of n=7 WT DEN+CD, n=16 WT DEN+HFD, n=7 *Il1r1* ^Hep−/−^ DEN+CD, n=16 *Il1r1* ^Hep−/−^ DEN+HFD mice at 24 weeks of age. Data in E represent represent mean±SEM of n=7 WT DEN+CD, n=14 WT DEN+HFD, n=7 *Il1r1* ^Hep−/−^ DEN+CD, n=14 *Il1r1* ^Hep−/−^ DEN+HFD mice at 24 weeks of age. *p<0.05 for WT versus *Il1r1* ^Hep−/−^, and ^$^<0.05, ^$$^ *p*<0.01, ^$$$^ *p*<0.001 for DEN+CD versus DEN+HFD using Kruskal-Wallis H test followed by pairwise Bonferroni-corrected Mann-Whitney *U* tests (A–C) or one-way ANOVA with post hoc Tukey tests (E). Abbreviations: CD, control diet; DEN, diethylnitrosamine; HFD, high-carbohydrate diet; WT, wild type.

To assess liver tumor formation, the number and size of macroscopically detectable tumor nodules on the liver surface of all lobes were counted. The incidence of macroscopic tumor development was 100% in both genotypes after week 18 of HFD feeding, whereas 57% (4/7) of WT and 29% (2/7) of *Il1r1* ^Hep−/−^ mice in the corresponding DEN+CD control groups presented solitary small tumor nodules. The average number of liver nodules was significantly higher in the DEN+HFD group than in the DEN+CD group, which confirmed the strong tumor-promoting effect of HFD (Figure 3A).^24,25,26^ Strikingly, HFD-fed *Il1r1* ^Hep−/−^ mice developed 43% less tumors compared to WT littermates (13.3±1.9 vs. 23.0±3.7, *p*=0.08, Figure 3A), with a significant reduction of tumor nodules >1 mm (3.0±0.6 vs. 7.8±1.7, *p*<0.05, Figure 3B). Histological analysis showed that almost all lesions arising in DEN-injected mice after 18 weeks of feeding were dysplastic foci/nodules, whereas sections from DEN+HFD–treated *Il1r1* ^Hep−/−^ mice showed fewer and smaller-sized liver lesions than those from WT mice (Figure 3B). Remarkably, 19% (3/16) of the WT mice developed tumor nodules even >10 mm in diameter. This translated into a significant reduction of tumor load in *Il1r1* ^Hep−/−^ mice compared to WT littermates (39.4±5.9 vs. 89.5±17.4 mm^2^, *p*<0.05, Figure 3C). Furthermore, the hepatic expression of Ki-67, which is strongly associated with tumor cell proliferation and growth, seemed to be increased in WT steatotic livers than in those from *Il1r1* ^Hep−/−^ mice (Figure 3D), whereas quantitative real-time-PCR analysis detected similar gene expression levels of Ki-67 and the proto-oncogene cyclin D1 in both genotypes (Figure 3E). These results suggest that IL-1/IL-1R1 signaling in hepatocytes is an important contributing factor to DEN-initiated steatosis-related hepatocarcinogenesis, and blocking this pathway slows down liver tumor growth in obese mice. The relevance of the hepatocyte IL-1R1 for HCC could also been shown in another NASH-associated in vivo model of hepatocarcinogenesis. Mice received low doses of profibrogenic carbon tetrachloride for 12 weeks and were fed a western or CD, respectively. WT mice developed dysplastic nodules with >1 mm at significantly higher rates than *Il1r1* ^*Hep−/−*^ mice (62.5% [5/8] vs. 28.6% [2/7]), while liver histology in terms of steatosis, inflammation, and fibrosis did not differ (Supplemental Figure S1, http://links.lww.com/HC9/B79).

### IL-1R1 is involved in the acute cell death response induced by DEN

IL-1R1 expression did not affect the hepatocyte cell death rate in mice exposed to DEN+HFD since serum parameters for liver toxicity, ALT, AST, LDH (all Figure 2A), hepatic MDA levels (Figure 2F), and the hepatic activity of caspase 3 were similar in the 2 genotypes at 24 weeks of age. This suggests that IL-1R1 might not act on hepatocyte cell death during tumor progression but during initiation or early tumor promotion. To reveal the basis for decreased liver tumor susceptibility, we examined the acute effects of DEN administration on liver enzymes in 4- and 6-week-old *Il1r1* ^Hep−/−^ and WT mice before the start of dietary feeding (Table 1). At 4 and 6 weeks of age, ALT levels were in the normal range, whereas AST levels were comparably elevated in both genotypes at 4 weeks of age. AST levels decreased to almost normal levels in *Il1r1* ^Hep−/−^ mice at 6 weeks of age, whereas WT mice displayed elevated AST levels. Furthermore, serum levels of LDH were still increased in WT mice at 6 weeks of age, whereas there was a trend for lower LDH levels in *Il1r1* ^Hep−/−^ mice. These data indicate prolonged DEN-induced hepatocellular injury early following tumor initiation in WT compared to *Il1r1* ^Hep−/−^ livers and the involvement of IL-1/IL-1R1 signaling in the acute cell death response induced by DEN. In line with this, a caspase 3 enzyme assay in whole liver tissue homogenates validated a significantly higher rate of hepatocyte apoptosis in WT versus *Il1r1* ^Hep−/−^ mice at week 2 post-DEN (Table 1). However, within 4 weeks after DEN injection, this effect decreased, and there was no difference in hepatic caspase 3 activity between 6-week-old *Il1r1* ^Hep−/−^ and WT mice. Similarly, the increased sensitivity of WT hepatocytes to DEN was evident through the lower hepatic activity of cytoprotective NF-κB p65 in mice at week 4 post-DEN (*p*=0.067, Table 1).

**TABLE 1:** Liver serum parameters, hepatic caspase 3 and NF-κB p65 activities in *Il1r1* ^Hep−/−^ and WT mice at week 2 and 4 post-DEN injection

	4 wk		6 wk	
	WT DEN	*Il1r1* ^Hep−/−^ DEN	WT DEN	*Il1r1* ^Hep−/−^ DEN
ALT (U/L)	24±0	24±0	24±0	24±0
AST (U/L)	116±23	112±11	121±15	75±7^a^
LDH (U/L)	500±87	599±65	513±101	349±83
Relative hepatic caspase 3 activity	1.00±0.07	0.53±0.13^a^	0.50±0.14	0.50±0.17
Relative hepatic NF-κB p65 activity	1.00±0.13	0.81±0.10	1.00±0.04	1.20±0.08

*Note:* Serum ALT, AST, and LDH levels from overnight-fasted *Il1r1* ^Hep−/−^ and WT mice and relative hepatic caspase 3 and NF-κB p65 activities were assessed at weeks 2 and 4 post-DEN. (Data are displayed as the mean±SEM of n=5 WT DEN and n=8 *Il1r1* ^Hep−/−^ DEN mice at 4 weeks of age and n=7 WT DEN and n=6 *Il1r1*^Hep−/−^ DEN mice at 6 weeks of age).

^a^ *p*<0.05, for WT versus *Il1r1*^Hep−/−^ using the Mann-Whitney *U* test (AST) or unpaired, two-tailed Student *t* test (hepatic caspase 3 activity).

Abbreviations: DEN, diethylnitrosamine; LDH, lactate dehydrogenase; WT, wild type.

**FIGURE 4:**
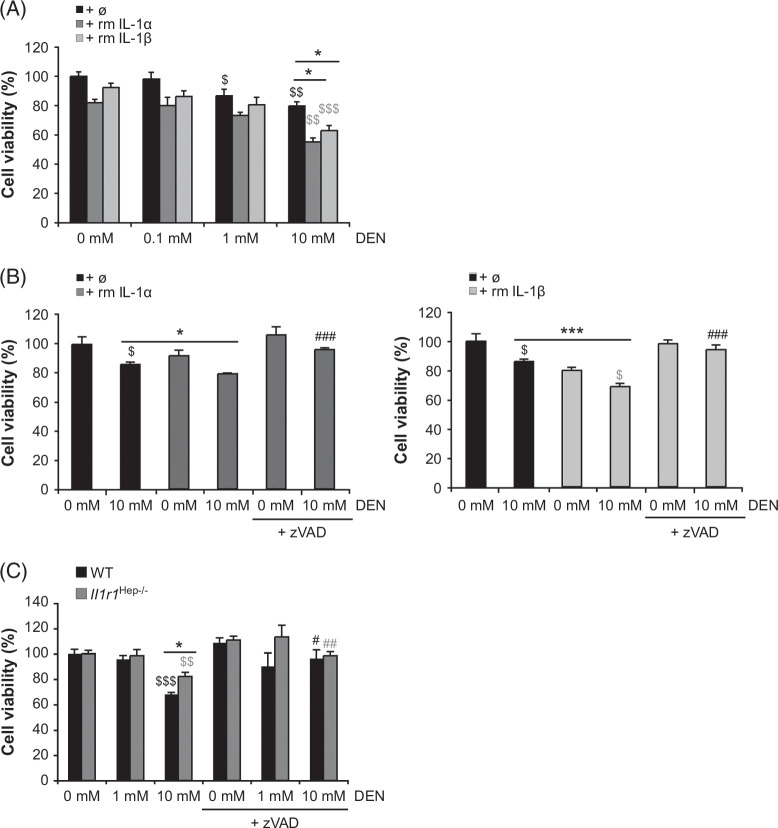
Rate of DEN-induced hepatocyte cell death in vitro. (A and B) Primary WT hepatocytes were treated ex vivo with different concentrations of DEN (0–10 mM) in the absence or presence of rm IL-1α or rm IL-β protein (10 ng/mL). Pan-caspase inhibitor zVAD (50 µM), wherever indicated, was added 1 hour before DEN treatment (B). After 24 hours, cell viability was assessed by MTT colorimetric assay relative to untreated samples. (C) The hepatotoxic effects of DEN and the involvement of caspases in hepatocellular death were compared between primary WT and *Il1r1* ^Hep−/−^ hepatocyte cultures. (Numerical data in mean±SEM of 3 (A and C) or 2 (B) independent experiments performed in at least duplicate readings. * *p*<0.05, *** *p*<0.001 for—rm IL-1α/β versus + rm IL-1α/β (A and B) or WT versus *Il1r1* ^Hep−/−^ (C), ^$^ *p*<0.05, ^$$^ *p*<0.01, ^$$$^ *p*<0.001 for—DEN versus + DEN, and ^#^ *p*<0.05, ^##^ *p*<0.01, ^###^ *p*<0.001 for—zVAD versus + zVAD according to an unpaired, two-tailed Student *t* test.). Abbreviations: DEN, diethylnitrosamine; rm, recombinant mouse; zVAD, pan-caspase inhibitor.

In agreement with this, in vitro studies demonstrated increased susceptibility of WT hepatocytes to DEN-induced acute toxicity in the presence of recombinant IL-1α or IL-1β protein (Figure 4A). DEN-induced hepatocyte cell death in the presence of recombinant IL-1α/β protein was responsive to caspase inhibition by the pan-caspase inhibitor zVAD (Figure 4B and C). In contrast, the hepatotoxic effects of DEN were significantly reduced in *Il1r1* ^Hep−/−^ hepatocytes (Figure 4C), suggesting that IL-1/IL-1R1 signaling facilitates apoptosis in stressed hepatocytes, as previously described for metabolic stress conditions.^11^ In conclusion, only a small fraction of all hepatocytes undergo cell death in response to a carcinogenic dose of DEN, and apoptosis might be enhanced by IL-1/IL-1R1 signaling, triggering a more pronounced compensatory proliferation and malignant progression of surviving cells,^12,27^ which is aggravated in HFD-fed mice.^24^

### Aberrant activation of hepatic JNK and STAT3 is downregulated in the steatotic liver of Il1r1^Hep−/−^ mice

**FIGURE 5:**
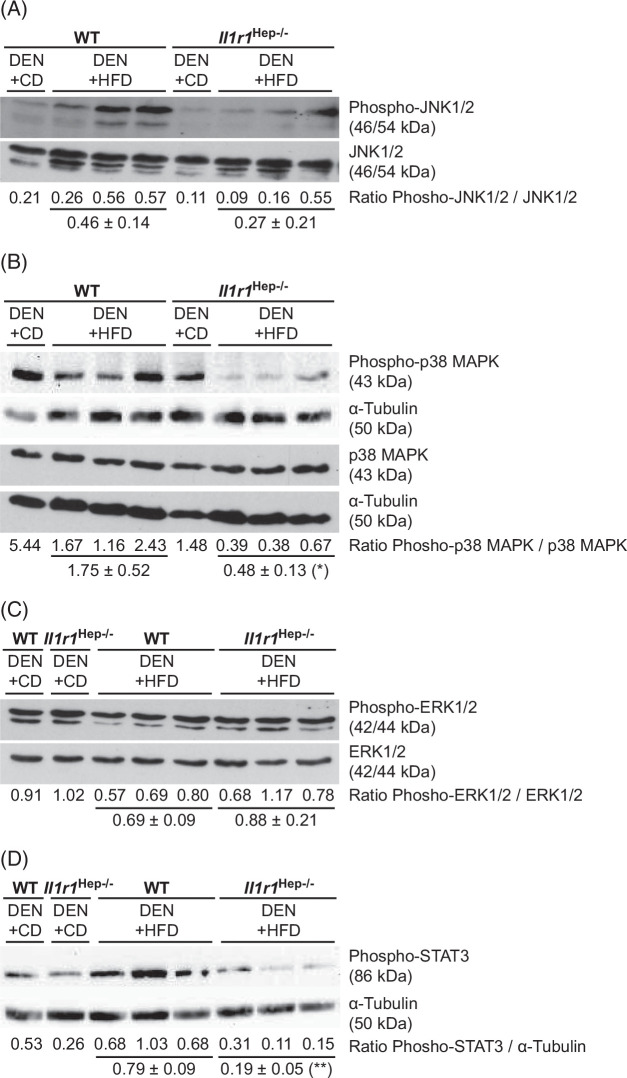
DEN+HFD-induced alterations of MAPK signaling and phospho-STAT3 levels in the liver of *Il1r1* ^Hep−/−^ and WT mice. Immunoblotting of (A) phospho-JNK (Thr183/Tyr185), (B) phosho-p38 MAPK (Thr180/Tyr182), (C) phospho-ERK1/2 (Thr202/Tyr204), and total (A) JNK, (B) p38 MAPK, and (C) ERK1/2 protein, and (D) phospho-STAT3 (Tyr705) in tumors surrounding liver tissue lysates of the different experimental groups after DEN injection and 18 weeks of HFD or CD feeding. (In A–D representative immunoblots with densitometric analysis are shown. Total MAPK proteins resp. α-tubulin served as protein loading control. *p<0.05, ** *p*<0.01 for WT versus *Il1r1* ^Hep−/−^ according to an unpaired, two-tailed Student *t* test (B and D). Uncropped images from western blots are shown in Supplemental Figure S2, http://links.lww.com/HC9/B79.). Abbreviations: CD, control diet; DEN, diethylnitrosamine; ERK, extracellular signal-regulated kinases; HFD, high-carbohydrate diet; JNK, rm, recombinant mouse; WT, wild type; zVAD, pan-caspase inhibitor.

Differences in the signal transduction pathways responsible for the enhanced survival and proliferation of malignant hepatocytes might underlie the reduced tumor growth in HFD-fed *Il1r1* ^Hep−/−^ mice compared to WT littermates. Due to their relevance in MASLD and cancer, we analyzed the phosphorylation state of stress-activated kinases JNK1/2, p38, and ERK1/2 in tumors surrounding liver tissue homogenates from *Il1r1* ^Hep−/−^ and WT mice by western blotting (Figure 5A–C). Consistent with previous findings, WT mice treated with DEN+HFD exhibited elevated JNK1/2 activity in the liver at week 18 of feeding, which was less pronounced in *Il1r1* ^Hep−/−^ littermates (2-fold).^11^ In parallel, we detected a marked decrease in phosphorylated p38 levels in the liver of both DEN+HFD groups compared to the DEN+CD groups, whereby ablation of IL-1R1 additionally reduced p38 phosphorylation in lean and obese mouse livers by 4-fold. In contrast, the protein levels of total and phosphorylated ERK1/2 were not altered by the genotype or treatment.

Furthermore, liver tissue from DEN+HFD–treated WT mice displayed markedly increased Tyr705-phosphorylation of STAT3), indicative of STAT3 activation (Figure 5D), which can stimulate the proliferation and progression of malignantly transformed hepatocytes.^28^ Interestingly, hepatic phospho-STAT3 levels were significantly lower in obese *Il1r1* ^Hep−/−^ mice than in their WT littermates. These findings indicate that IL-1/IL-1R1 signaling contributes to hepatic JNK and STAT3 activation under HFD conditions, which may favor DEN-initiated liver tumor growth.

### Inhibition of hepatic IL-1R1 signaling prevents early infiltration of CD11b^+^Ly6C^high^Ly6G^−^ cells and CD8^+^ T cells in the liver of HFD-fed mice

Since immune cells impact the development and progression of liver tumors in the microenvironment, we analyzed intrahepatic immune cell composition using flow cytometry (Table 2). In both genotypes, HFD augmented the total numbers of liver macrophages (CD45^+^F4/80^+^) together with an increase of CD86 expression, indicating a pronounced inflammatory M1 polarity. Further, we detected a shift from CD45^+^CD11b^+^Ly6C^low^ to ^high^Ly6G^-^F4/80^+^ cells in *Il1r1* ^Hep−/−^ mice on CD compared to kockouts on HFD. This suggests that monocyte-derived macrophages are recruited to the liver to complement the macrophage pool in response to DEN- and HFD-induced liver injury. Furthermore, CD86 was slightly upregulated, whereas the anti-inflammatory marker CD206 was downregulated in the DEN+HFD group, suggesting a macrophage polarization from an anti-inflammatory phenotype M2 to a proinflammatory M1 phenotype. Remarkably, we observed an increased percentage of CD45^+^CD11b^+^Ly6C^high^Ly6G^-^F4/80^−^ cells in the WT DEN+HFD group. Ly6C^high^ monocytes have been shown to be precursors of monocytic myeloid-derived suppressor cells, which promote tumor growth and suppress immune cell functions in the microenvironment.^29,30,31^ In contrast, granulocytic CD45^+^CD11b^+^Ly6C^−^Ly6G^high^ cells were not affected by intervention or genotype. By studying intrahepatic lymphocyte populations, we noticed an early loss of intrahepatic CD4^+^ T cells (CD45^+^CD3^+^CD4^+^) and regulatory CD4^+^CD25^+^ T cells (CD45^+^CD3^+^CD4^+^CD25^+^) in HFD-fed mice, irrespective of IL-1R1. CD8^+^ T cells (CD45^+^CD3^+^CD8^+^) accumulated in WT steatotic livers, whereas this infiltration was absent in *Il1r1* ^Hep−/−^ mice. In contrast, the relative number of hepatic NK cells (CD45^+^CD3^-^NK1.1^+^) appeared to comparably increase after HFD feeding in both genotypes. The proportion of B cells did not vary significantly between groups. Taken together, these data indicate that the reduced tumor growth in DEN+HFD–treated *Il1r1* ^Hep−/−^ mice was associated with reduced accumulation of intrahepatic CD11b^+^Ly6C^high^Ly6G^−^ cells and CD8^+^ T cells.

**TABLE 2:** Flow cytometry analysis of intrahepatic immune cells in *Il1r1* ^Hep−/−^ and WT mice treated with DEN+HFD and DEN+CD controls

Intrahepatic immune cells (live^+^, CD45^+^)	WT DEN+CD	*Il1r1* ^Hep−/−^ DEN+CD	WT DEN+HFD	*Il1r1* ^Hep−/−^ DEN+HFD
F4/80^+^ (%)^a^	10.8±1.2	12.8±2.4	14.9±1.4	16.1±1.5
^a^MFI CD86	914±90	825±3	1050±135	1255±135^b^
CD11b^+^Gr-1^+^/Ly6C^low^ to ^high^ Ly6G^−−^F4/80^+^ (%)^a^	4.1±1.9	2.2±0.1	3.6±0.6	3.8±0.7
^a^MFI CD86	1076±93	910±51	1082±63	1337±233
^a^MFI CD206	4635±1068	3544±729	2496±149 (c)	2455±117
CD11b^+^Gr-1^+^/Ly6C^high^Ly6G^−^F4/80^−^ (%)^a^	11.0±1.0	11.8±1.3	14.6±1.8	11.6±1.4
^a^MFI CD86	277±7	327±23	404±36^b^	361±21
CD11b^+^Gr-1^+^/Ly6C^−^Ly6G^+^F4/80^−^ (%)	0.8±0.3	1.1±0.3	0.8±0.2	1.0±0.3
CD3^+^CD4^+^ (%)	10.7±1.2	13.2±1.4	8.9±0.6	9.1±0.6^c^
CD3^+^CD4^+^CD25^+^ (%)	4.3±0.7	5.8±0.8	3.1±0.3	3.2±0.3^c^
CD3^+^CD8^+^ (%)	6.6±0.4	8.6±0.6	9.5±1.2^a^	6.7±0.5
CD3^-^CD45R^+^ (%)	48.5±2.2	41.3±2.9	43.9±2.2	47.5±2.7
CD3^−^CD45R^−^NK1.1^+^ (%)	4.6±0.6	5.0±0.9	7.7±0.9	7.2±0.9

*Note:* Intrahepatic immune cells were isolated from tumors surrounding liver tissue of the different experimental groups and CD45^+^ cell subpopulations were analyzed by flow cytometry. (Data represent mean±SEM of n=7 WT DEN+CD, n=16 WT DEN+HFD, n=7 *Il1r1* ^Hep-/-^ DEN+CD, and n=16 *Il1r1*^Hep-/-^ DEN+HFD mice at 24 weeks of age.)

^a^ *p*<0.05, for WT versus *Il1r1*^Hep−/−^.

^b^ *p*<0.05.

^c^ *p*<0.01, for DEN+CD versus DEN+HFD using two-way ANOVA followed by Bonferroni multiple comparisons tests.

Abbreviations: CD, control diet; DEN, diethylnitrosamine; HFD, high-carbohydrate diet; MFI, mean fluorescence intensity; WT, wild type.

Owing to differences in immune cell composition, we also analyzed hepatic cytokine and chemokine expression (Supplemental Table S4, http://links.lww.com/HC9/B79). We found marked increases in multiple cytokines and chemokines, including IL-1α/β, IL-1R antagonist, IL-10, interferon-γ, TGF-β, MCP-1, and CXCL1/2 mRNA levels in the liver tissue from DEN in the 2 genotypes, especially after HFD feeding, whereas the levels of TNF-α and IL-6 were only slightly affected by DEN+HFD. The most striking differences between genotypes were observed for CXCL1 and CXCL2. However, the increase in both chemokines in WT mice relative to *Il1r1* ^Hep−/−^ mice was not significant. In general, we found no difference between serum (Figure 1F) and hepatic cytokine/chemokine levels (Supplemental Table S4, http://links.lww.com/HC9/B79) between *Il1r1* ^Hep−/−^ and WT mice, which might indicate, above all, intrinsic IL-1R1-dependent alterations in tumor cells. However, even minor changes in the local microenvironment may affect chronic inflammation, which supports tumor growth at later stages.

### Prolonged HFD feeding induces onset of HCC in Il1r1^Hep−/−^ and WT mice, but with reduced tumor sizes in Il1r1^Hep−/−^ steatotic livers

To examine hepatocarcinogenesis at a later stage, we prolonged the HFD feeding for up to 24 weeks. The dysmetabolic phenotype of *Il1r1* ^Hep−/−^ and WT mice was hardly affected by prolonged HFD feeding (Supplemental Figure S3, http://links.lww.com/HC9/B79), whereas the liver phenotype was aggravated irrespective of IL-1R1, as indicated by marked increases in serum transaminase, LDH, and AFP levels, more severe hepatomegaly, and histological disease activity in *Il1r1* ^Hep−/−^ and WT mice (Supplemental Figure S4, http://links.lww.com/HC9/B79). Similarly, the formation and growth of DEN-induced liver tumors were significantly increased in the 2 genotypes. However, the total number of tumors developing in the *Il1r1* ^Hep−/−^ steatotic livers was still reduced by about 30% compared to the WT (55.5±10.1 vs. 79.1±9.2, n.s., Supplemental Figure S5A, http://links.lww.com/HC9/B79), whereby the average number of large liver nodules (>5 mm in diameter) was significantly smaller in the *Il1r1* ^Hep−/−^ DEN+HFD group compared to the WT DEN+HFD group (1.8±0.7 vs. 5.3±1.2, *p*<0.05, Supplemental Figure S5B, http://links.lww.com/HC9/B79). This was also reflected by a lower tumor load in *Il1r1* ^Hep−/−^ relative to WT hepatic tissue (306.8±76.5 vs. 563.0±97.0 mm^2^, *p*=0.09, Supplemental Figure S5C, http://links.lww.com/HC9/B79). Histopathological analysis confirmed the presence of HCCs and/or mixed HCC/intrahepatic cholangiocarcinoma in 62% (10/16) of WT and 67% (8/12) of *Il1r1* ^Hep−/−^ mice maintained on an HFD for 24 weeks. Importantly, the determination of the ratios of the tumor area to the whole histological section area by digital means confirmed the protective effects of IL-1R1 knockout on liver tumor growth (Supplemental Figure S5D, http://links.lww.com/HC9/B79). Likewise, gene expression analysis revealed reduced expression of Ki-67 and cyclin D1 in tumors surrounding liver tissue from *Il1r1* ^Hep−/−^ mice, as well as of cyclin D1 in individual tumors from *Il1r1* ^Hep−/−^ mice compared to those from WT mice (Supplemental Figure S5E, http://links.lww.com/HC9/B79). Overall, these results showed that blocking the IL-1R1 signaling pathway in hepatocytes did not prevent malignant transformation and tumor formation in the DEN+HFD model but substantially suppressed tumor growth in obese mice at a later stage. Interestingly, under CD conditions, macroscopic tumor development occurred in 100% (8/8) of WT mice, but only 57% (4/7) of *Il1r1* ^Hep−/−^ mice at 30 weeks of age, and was paralleled by pronounced increases in serum ALT, AST, LDH, AFP levels, and liver weights in the WT DEN+CD group (Supplemental Figure S4 and S5, http://links.lww.com/HC9/B79). This further supports the beneficial effects of hepatic IL-1R1 inhibition with respect to tumor formation, which might be, at least in part, overridden by chronic metabolic inflammation induced by HFD.

## DISCUSSION

An important liver-related complication of MASLD, even in the absence of cirrhosis, is HCC development.^2^ Previously, IL-1-targeted approaches to block the cytokine, its receptor, and downstream signaling have shown benefits in obesity-associated insulin resistance and liver injury.^11^ In addition, IL-1 influences the tumor microenvironment and tumor cells during cancer progression. Therefore, we investigated whether blocking the IL-1R1 signaling pathway in hepatocytes could prevent tumor formation in steatotic livers by employing transgenic *Il1r1* ^Hep−/−^ mice and their WT littermates^18^ using the DEN+HFD model.^25^ Our data revealed that targeted knockout of IL-1R1 in hepatocytes did not protect mice from malignant transformation of liver cells in response to DEN and HFD feeding but significantly slowed tumor growth in steatotic livers.

One finding underlying the decreased hepatocarcinogenesis in *Il1r1* ^Hep−/−^ mice could be linked to less severe DEN-induced hepatocellular injury following tumor initiation in these mice. We observed lower levels of transaminases, LDH, and hepatic caspase 3 activity in *Il1r1* ^Hep−/−^ mice than in WT mice. Using ex vivo hepatocyte cultures, we showed that hepatocytic IL-1R1 acts as an amplifier of hepatocyte death in response to DEN-induced cell stress, as previously observed under other stress conditions.^11,18^ Interestingly, a global IL-1R1 knockout suggested that hepatocyte necrosis triggered IL-1α release-mediated DEN-induced compensatory proliferation and liver tumorigenesis through IL-1R1/MyD88 signaling in KCs, with subsequent IL-6 release.^12^ Based on our data, we hypothesize that the IL-1R1 signaling pathway in hepatocytes acts as an important amplifier in this scenario. This also explains the reduced tumor load observed in DEN-injected *Il1r1* ^Hep−/−^ mice under CD conditions. Furthermore, although HFD is protumorigenic^26^ and led to a 100% incidence of dysplastic foci/nodules in the liver tissue of all DEN-challenged mice at week 18, IL-1R1 knockout resulted in fewer and significantly smaller tumor nodules and reduced Ki-67 staining in the liver tissue, indicating reduced proliferative activity. In contrast, surrogate markers of liver cell damage did not differ significantly between genotypes at this phase of tumor development.

The reduced susceptibility of Il1r1Hep−/− mice to HFD-driven liver tumor growth compared to WT mice may be linked to improvements in insulin resistance/hyperinsulinemia and hepatic steatosis. Both findings are in agreement with observations from our previous study showing improved whole-body and hepatic insulin sensitivity through sustained mitochondrial function in *Il1r1* ^Hep−/−^ mice.^11^ The effects of dietary intervention and genotype on the expression levels of all examined genes associated with metabolism were only mild in the DEN+HFD model, the trends for peroxisome proliferator-activated receptor-α, carnitine palmitoyltransferase 1, farnesoid X receptor-α, and in particular HO-1, however, coincided with the results of our previous study. HO-1 plays several important roles in hepatocytes, including anti-inflammatory, antiapoptotic, and antioxidant activities.^32^ In mice, HO-1 induction can prevent fibrosis progression by inducing an antioxidant pathway.^33^ Furthermore, in vitro and in vivo data have shown that HO-1 induction attenuates the proliferation, migration, and invasion of human HCC cells by suppressing IL-6 and phosphorylation of p38 MAPK and cyclin D1.^34^ In agreement with this, *Il1r1* ^Hep−/−^ mice also exhibited decreased phospho-p38 levels in liver tissue as well as lower cyclin D1 expression in both tumors surrounding liver tissue and individual tumors after HFD feeding.

Cyclin D1 is a proto-oncogene that is intimately involved in abnormal cell growth processes, angiogenesis, and resistance to apoptosis and favors aggressive liver tumor progression. Functional studies by Wu et al^35^ revealed that autophagy-selective degradation of cyclin D1 plays a suppressive role in cell proliferation and liver tumor formation. This is an interesting point, as *Il1r1* ^Hep−/−^ mice showed improved autophagy status in the steatotic liver compared to WT littermates in the 12-week HFD model.^11^ Luo and colleagues observed that hyperinsulinemia maintains the heightened hepatic expression of cyclin D1 in various models of obesity/diabetes. Liver-specific cyclin D1 deficiency protects obese/diabetic mice against hepatic tumorigenesis, whereas lean/nondiabetic mice develop tumors, irrespective of cyclin D1 status.^36^ Thus, despite HFD feeding, the insulin-sensitive state of *Il1r1* ^Hep−/−^ mice, including reduced insulin levels, might contribute to the reduced cyclin D1 status, which slows down liver tumor growth under these conditions.

To adapt to HFD-induced metabolic stress, JNK was activated in WT steatotic livers but not in *Il1r1* ^Hep−/−^ mice. We recently showed that IL-1/IL-1R1 signaling in hepatocytes is a potent inducer of JNK signaling in MASLD.^11^ Sustained activation of JNK attenuates β-oxidation and favors hepatic steatosis and insulin resistance.^37^ JNK1, rather than JNK2, was found to promote these features.^38^ Furthermore, there is ample evidence that JNK1 is the main player in HCC pathogenesis.^39^ Activated JNK promotes cell cycle progression.^40^ Importantly, IL-1β-induced JNK activation plays a role in the upregulation of the oncoprotein Gankyrin in HCC.^14^ Thus, reduced hepatic JNK activity in obese *Il1r1* ^Hep−/−^ mice could reduce tumor growth in response to DEN+HFD.

Likewise, we found reduced hepatic STAT3 activation in obese *Il1r1* ^Hep−/−^ mice compared to that in WT mice. STAT3 is activated primarily by cytokines, particularly IL-6, growth factors, and similar to JNK, in response to oxidative stress.^28^ We can only speculate that increased HO-1 expression in DEN+HFD–treated *Il1r1* ^Hep−/−^ mice might counteract the activation of STAT3. Recently, we found that *Il1r1* ^Hep−/−^ mice express SOCS3 at elevated levels compared to their WT littermates under HFD conditions,^11^ which may also contribute to the dephosphorylation of STAT3. STAT3 activation is detected in HCC cells and is essential for the exacerbation of DEN-induced HCC in obesity.^25^ Furthermore, STAT3 activation contributes to impaired effectiveness of immune surveillance against HCC.^41^ We also observed differences in the intrahepatic immune milieu between *Il1r1* ^Hep−/−^ and WT mice in response to DEN+HFD. In WT steatotic livers, we detected an increase in CD45^+^CD11b^+^Ly6C^high^Ly6G^−^F4/80^−^ cells, which have been shown to be precursors of myeloid-derived suppressor cells, promoting tumor growth and suppressing immune cell functions in the microenvironment.^29,31,42^ These cells are recruited through CXCL1 and CXCL2, which are produced by hepatocytes under inflammatory conditions in response to IL-1α/β.^11^ Likewise, tumor cells and tumor-infiltrating CD11b^+^ myeloid cells can contribute to the high expression of CXCL1 and CXCL2, and deletion of CXCL1 and CXCL2 delays in vivo tumor growth.^43^ Additionally, CXCL1 and CXCL2 have been shown to promote the resistance of HCC cells to sorafenib,^44^ whereas C-X-C motif chemokine receptor 2 inhibition induces reprogramming of the tumor immune environment that promotes immune checkpoint inhibition in MASH-HCC.^45^ Interestingly, we detected increasing numbers of CD8^+^ T cells from DEN+HFD only in the WT, while expression levels of TNF-α and interferon-γ were reduced. CD8^+^ T cells have been identified as key drivers of hepatic insulin resistance^46^ and the progression of MASH, together with NKT cells, plays a pivotal role in MASH-to-HCC progression through the secretion of proinflammatory molecules and nonspecific killing of hepatocytes.^47,48^ Importantly, the progressive accumulation of dysfunctional/exhausted PD-1^+^ CD8^+^ T cells during MASH, for example, through tumor-derived immunosuppressive exosomes,^49^ was recently shown to lead to impaired immune surveillance and the progression of MASH-driven HCC.^50^

In summary, blocking the IL-1R1 signaling pathway in hepatocytes not only improves the MASLD phenotype and insulin resistance, as previously demonstrated but also significantly slows hepatocarcinogenesis in the context of hepatic steatosis. These findings suggest that IL-1R1 inhibition could be a promising strategy to reduce HCC risk in patients with MASLD and may serve as an adjunctive approach in HCC immunomodulation. Future studies should explore the clinical potential of this approach, particularly in high-risk populations, and further elucidate the underlying molecular mechanisms.

## Supplementary Material
