# Supplementary material for: Blocking interleukin-1 receptor type 1 (IL-1R1) signaling in hepatocytes slows down diethylnitrosamine-induced liver tumor growth in obese mice
Source: Hepatol Commun. 2024 Nov 29;8(12):e0568. doi: 10.1097/HC9.0000000000000568 (PMC11608749; doi:10.1097/HC9.0000000000000568)
Supplement: Supplementary file 1 [file hc9-8-e0568-s001.pdf]

Supplementary Data

Blocking interleukin-1 receptor type 1 (IL-1R1) signaling in hepatocytes slows down diethylnitrosamine-induced liver tumor growth in obese mice

Nadine Gehrke[1], Lea J. Hofmann[1], Beate K. Straub[2], Dirk A. Ridder[2], Ari Waisman[3,4], Leonard Kaps[5,6], Peter R. Galle[1,4], Jörn M. Schattenberg[1, 5, 7]

[1]I. Department of Medicine, [2]Institute of Pathology, [3]Institute for Molecular Medicine, and [4]Research Center for Immunotherapy, University Medical Center of the Johannes Gutenberg University Mainz, Mainz, Germany, [5]Department of Internal Medicine II, Saarland University Medical, Homburg, Germany, [6]Department of Dermatology, University Medical Center of the Johannes Gutenberg University Mainz, Mainz, Germany, [7]Saarland University, Saarbrücken, Germany.

Supplementary Material and Methods

*DEN and carbon tetrachloride (CCl_4)/Western diet (WD)-induced hepatocarcinogenesis*For HCC induction, 2-week-old male transgenic (TG) *Il1r1*$^{Hep-/-}$ mice (Alb-Cre:IL-1R1$^{flox/flox}$) exhibiting a deletion of all signaling-capable IL-1R1 isoforms in hepatocytes (18) and their WT littermates received a single intraperitoneal (i.p.) injection of DEN (25 mg/kg body weight (BW) dissolved in phosphate-buffered saline (PBS), Sigma-Aldrich, Taufkirchen, Germany). 4 weeks later mice were randomly divided into two dietary groups, having free access to a high-fat diet (HFD, 35.5 % w/w crude fat (58 kJ%), metabolizable energy (ME): 5.45 kcal/g) and fructose/glucose (55/45 % w/v) enriched drinking water or a corresponding control diet (CD; 5.4 % w/w crude fat (13 kJ%), ME: 3.74 kcal/g) and plain water until sacrifice. The composition and energy density of the diets (both from ssniff Spezialdiäten GmbH, Soest, Germany) are listed in Supplementary Table S1. To control for baseline differences, age- and gender-matched mice of both genotypes received a single i.p. injection of PBS instead of DEN followed by CD feeding until sacrifice. Body weight and food consumption were tracked over the course of the feeding period. After 18 weeks of diet intervention, all mice were sacrificed after overnight fast (16- to 18-h) and blood and liver tissue were collected for further analyses. In separate experiments, male *Il1r1*$^{Hep-/-}$ mice and their WT littermates were sacrificed after 24 weeks of diet intervention to study the effects of prolonged HFD feeding on liver tumor growth, or already at week 2 or week 4 post DEN-injection before starting experimental feeding to study the acute effects of DEN on liver injury.

For the CCl_4/WD model, the protocol of Tsuchida et al. (J Hepatol., 2018) was used . 8-week-old, male *Il1r1*$^{Hep-/-}$ and WT mice were fed a WD containing 21.1 % fat, 41 % sucrose, and 1.25 % cholesterol by weight (ssniff Spezialdiäten GmbH) and a high sugar solution (23.1g/L d-fructose (Sigma-Aldrich, G8270) and 18.9 g/L d-glucose (Sigma-Aldrich, F0127)) or a corresponding control diet (ssniff Spezialdiäten GmbH) and plain water for 12 weeks. CCl_4 (Sigma-Aldrich, 289116-100ML) at the dose of 0.32 µg/g of BW was injected i.p. once/week, starting simultaneously with the diet administration for 12 weeks. The composition of the experimental diets (both from ssniff Spezialdiäten GmbH).

Analysis of hepatic tissue incl. macroscopic and microscopic tumor assessment

Tumor nodules on the liver surface of all lobes were counted after sacrifice. After weighing, individual tumors, tumor surrounding tissue and normal liver tissue were separated and asserved appropriately for further analysis. For histological examination of mouse liver tissue the left liver lobes were cut, fixed in 4 % paraformaldehyde-PBS, embedded in paraffin, and stained with hematoxylin & eosin (H&E) following standard procedures. Semiquantitative evaluation of steatosis ((0) no steatosis; (1) 1-10 %; (2) 11-50 %; (3) > 50 % of the hepatocytes with neutral fat deposition), lobular inflammation ((0) no inflammation; (1) little portal inflammation, 3–5 single necrotic cells/15 high-power fields (HPFs), no grouped necrotic cells; (2) intermediate portal inflammation, 6–9 single necrotic cells/15 HPFs and/or one focus of grouped necrotic cells; (3) severe portal inflammation, ≥ 10 single necrotic cells/15 HPFs and/or more than one focus of grouped necrotic cells), fibrosis ((0) no fibrosis; (1) fibrous portal expansion; (2) formation of fibrous septae), and tumor status was done blinded by an expert hepatopathologist (BKS). Immunhistochemical staining of Ki-67 was performed as previously described (22). Representative pictures were taken using an Olympus BX45 microscope (Olympus Deutschland, Hamburg, Germany) with a Jenoptik PROGRES GRYPHAX camera (Micro Optimal, Meerbusch, Germany) and the Olympus Image Analysis Software analySIS docu (Olympus Deutschland). To determine the ratios of tumor area per whole histological section area by digital means, the software Halo (Indica Labs, Albuquerque, NM, USA, Version 2.1.1637.11) was used.

Metabolic parameters

The following equations were used to determine HOMA-IR and Adipo-IR levels of the mice: HOMA-IR = [insulin level subsequent to fasting (µIU/ml) x glucose level following fasting (mg/dl) / 405] and Adipo-IR = [insulin level subsequent to fasting (pmol/l) x NEFA level following fasting (mmol/l)].

Statistical analysis

All statistical analysis was performed using GraphPad Prism 7 software (GraphPad Software, La Jolla, CA, USA). All results were initially submitted to Shapiro-Wilk normality test for normality and to Levene's test for homogeneity of variance. Comparisons between two experimental groups were carried out using the unpaired, two-tailed Student's *t* test or the Mann-Whitney *U* test to determine statistical significance of differences. Multiple experimental groups were analysed by two-way analysis of variance followed by Bonferroni multiple comparison post hoc test or Kruskal-Wallis H test followed by Mann-Whitney *U* test with a Bonferroni correction when the assumptions for ANOVA were not met. One-way ANOVA with post-hoc Tukey tests was used when multiple experimental groups including baseline controls were compared. The results with a p value of <0.05 were considered to be significant. All data are shown as mean ± standard error of mean (SEM) to determine the precision and differences of means and statistically significant values were assumed with $^{*/\$/\#}$ p<0.05, $^{**/\$\$/\#\#}$ p<0.01, $^{***/\$\$\$/\#\#\#}$ p<0.001. Statistical parameters are stated in the specific figure legends.

Supplementary Figure S1: CCl_4/WD-induced NASH and tumor development in *Il1r1*$^{Hep-/-}$ and WT mice.

Low dose weekly CCl_4 (i.p. 0.32 µg/g BW) was given for 12 weeks in 8-week-old, male *Il1r1*$^{Hep-/-}$ and WT mice.fed a high-fat, high-fructose und high-cholesterol western diet (WD, n=7-8 mice) in combination with fructose/glucose-enriched drinking water or a corresponding control diet (CD, n=5-7 mice) and plain water. At week 12 of intervention, mice were sacrificed and (A) absolute liver weight, (B) serum levels of ALT, (C) representative liver histology by H&E-staining (magnification: 10 x, scale bar: 100 µm) and pathological scores from 20-week-old *Il1r1*$^{Hep-/-}$ and WT mice after CCl_4/WD or CCl_4/CD treatment. (Data in A-C represent mean ± SEM of n= 7 WT CCl_4/CD, n= 5 *Il1r1*$^{Hep-/-}$ CCl_4/WD, n=8 WT CCl_4/WD, n= 7 *Il1r1*$^{Hep-/-}$ CCl_4/WD at 20 weeks of age. $^{\$}$ $p<0.05$, $^{\$\$}$ $p<0.01$, $^{\$\$\$}$ $p<0.001$ for CCl_4/WD vs. CCl_4/CD using two-way method of ANOVA followed by Bonferroni multiple comparisons tests (A) or Kruskal-Wallis H test followed by pairwise Bonferroni-corrected Mann-Whitney *U* tests (B and C). There was no statistically significant difference between *Il1r1*$^{Hep-/-}$ and WT mice.)

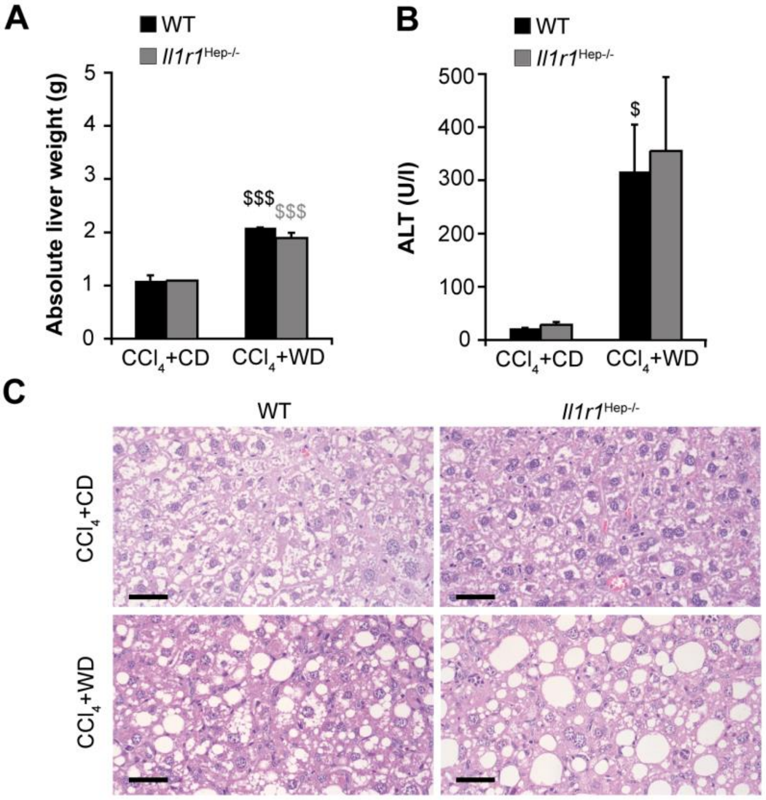

	WT CCl_4+CD	*Il1r1*$^{Hep-/-}$ CCl_4+CD	WT CCl_4+WD	*Il1r1*$^{Hep-/-}$ CCl_4+WD
**Steatosis (0-3)**	0.43 ± 0.17	0.20 ± 0.18	3.00 ± 0.0 ($$$)	3.00 ± 0.0 ($$$)
**Inflammation (0-3)**	1.14 ± 0.12	0.40 ± 0.22	2.00 ± 0.0 ($$)	2.00 ± 0.0 ($$)
**Fibrosis (0-3)**	0.29 ± 0.13	0.0 ± 0.0	1.00 ± 0.0 ($$)	1.00 ± 0.0 ($$)
**Foci (n)**	0.0 ± 0.0	0.0 ± 0.0	1.25 ± 0.52	0.50 ± 0.15

Supplementary Figure S2: Uncropped images from western blots. Uncropped western blot images are shown that correspond to Figure 5A (A), Figure 5B (B), Figure 5C (C), and Figure 5D (D).

A

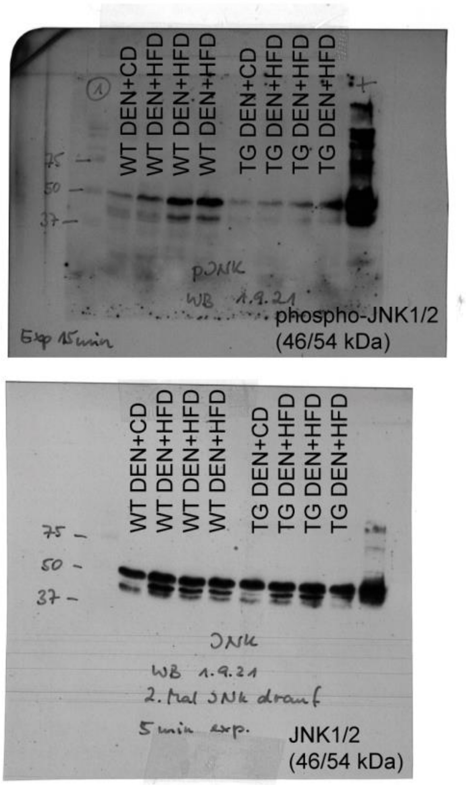

B

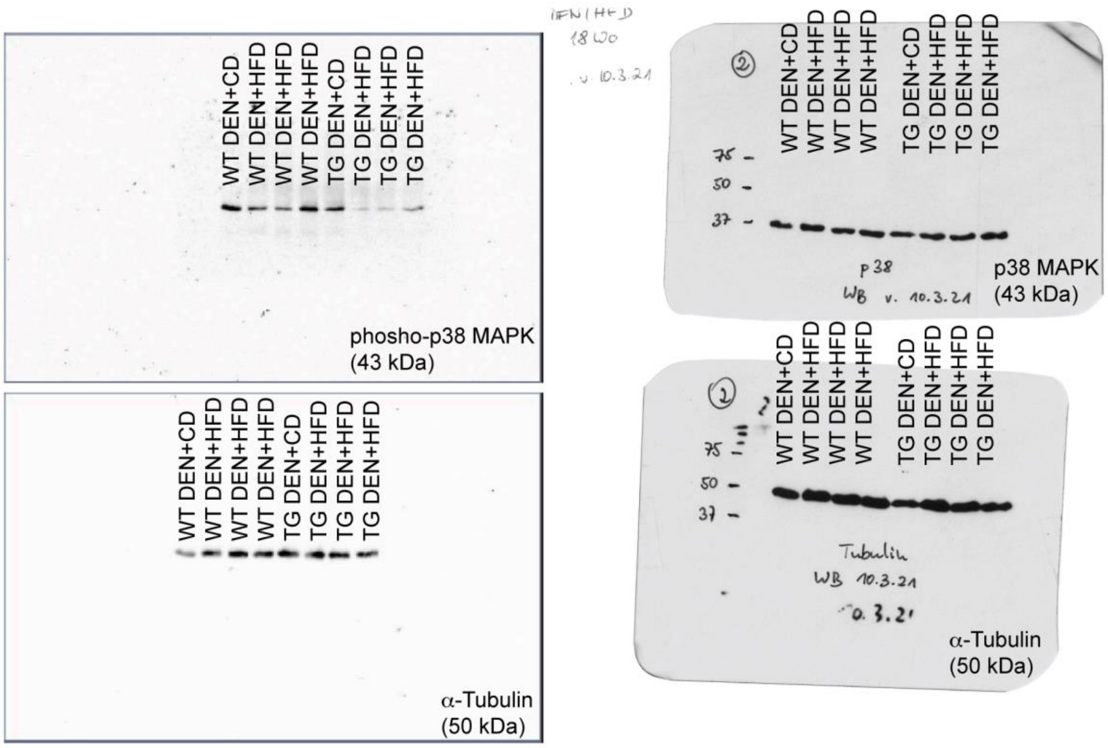

C

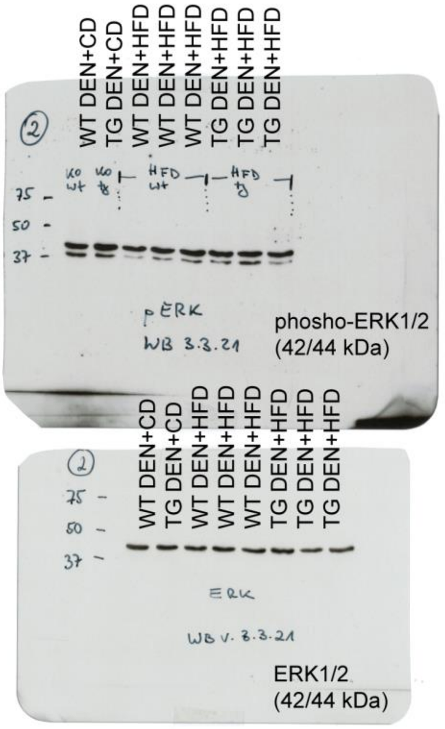
WT DEN+CD
TG DEN+CD
WT DEN+HFD
WT DEN+HFD
WT DEN+HFD
TG DEN+HFD
TG DEN+HFD
TG DEN+HFD
phosho-ERK1/2
(42/44 kDa)
WT DEN+CD
TG DEN+CD
WT DEN+HFD
WT DEN+HFD
WT DEN+HFD
TG DEN+HFD
TG DEN+HFD
TG DEN+HFD
ERK1/2
(42/44 kDa)

D

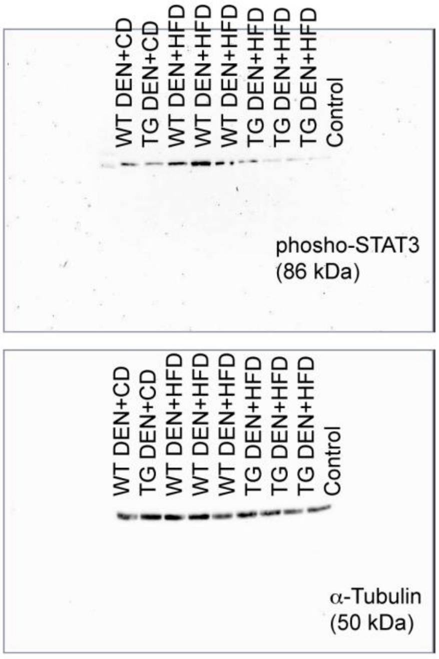
WT DEN+CD
TG DEN+CD
WT DEN+HFD
WT DEN+HFD
WT DEN+HFD
TG DEN+HFD
TG DEN+HFD
TG DEN+HFD
Control
phosho-STAT3
(86 kDa)
WT DEN+CD
TG DEN+CD
WT DEN+HFD
WT DEN+HFD
WT DEN+HFD
TG DEN+HFD
TG DEN+HFD
TG DEN+HFD
Control
α-Tubulin
(50 kDa)

Supplementary Figure S3: Body weight gain and systemic metabolic alterations in DEN-injected *Il1r1*$^{\text{Hep-/-}}$ and WT mice after prolonged HFD feeding. (A) Body weight curve and (B) mean caloric intake during the feeding period of 24 weeks. (C) At week 24 of feeding, sera from overnight-fasted *Il1r1*$^{\text{Hep-/-}}$ and WT mice were analyzed for levels of total cholesterol, triglycerides, glucose, insulin, and (D) HOMA-IR. (Data in A-D represent mean ± SEM of n=8 WT DEN+CD, n=7 *Il1r1*$^{\text{Hep-/-}}$ DEN+CD, n=16 WT DEN+HFD, n=12 *Il1r1*$^{\text{Hep-/-}}$ DEN+HFD mice at 30 weeks of age. * p<0.05 for WT vs. *Il1r1*$^{\text{Hep-/-}}$, and $^{\$}$ p<0.05, $^{\$\$}$ p<0.01, $^{\$\$\$}$ p<0.001 for DEN+CD vs. DEN+HFD using two-way method of ANOVA followed by Bonferroni multiple comparisons tests (A-C) or Kruskal-Wallis H test followed by pairwise Bonferroni-corrected Mann-Whitney *U* tests (C (insulin) and D).)

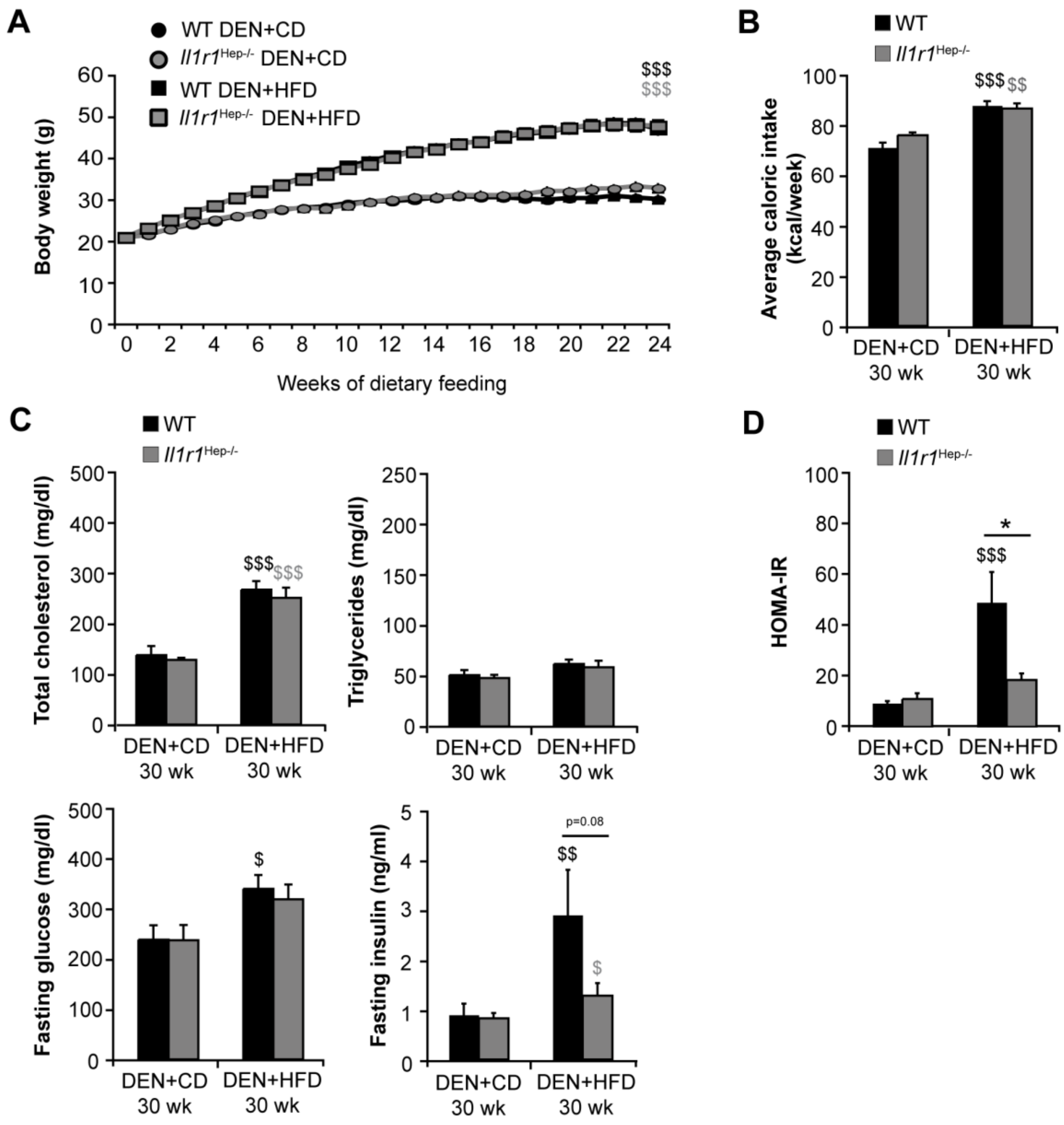

Supplementary Figure S4: Serum liver enzymes, AFP levels and MASLD grade in DEN-injected, HFD-fed *Il1r1*$^{Hep-/-}$ and WT mice at 30 weeks of age. (A) Serum levels of ALT, AST, LDH, and (B) AFP, (C) absolute liver weight, liver-to-body weight ratio, (D) representative macroscopic liver appearance, (E) representative liver histology by H&E staining (magnification: 10 x, scale bar: 100 µm) and pathological scores from 30-week-old *Il1r1*$^{Hep-/-}$ and WT mice after DEN+HFD or DEN+CD treatment. Data in A-C and E represent mean ± SEM of n=8 WT DEN+CD, n=7 *Il1r1*$^{Hep-/-}$ DEN+CD, n=16 WT DEN+HFD, n=12 *Il1r1*$^{Hep-/-}$ DEN+HFD mice at 30 weeks of age. $^{\$\$}$ p<0.01, $^{\$\$\$}$ p<0.001 for DEN+CD vs. DEN+HFD using Kruskal-Wallis H test followed by pairwise Bonferroni-corrected Mann-Whitney *U* tests (A and

E) or two-way method of ANOVA followed by Bonferroni multiple comparisons tests (B and C).

There was no statistically significant difference between *Il1r1*$^{Hep-/-}$ and WT mice.

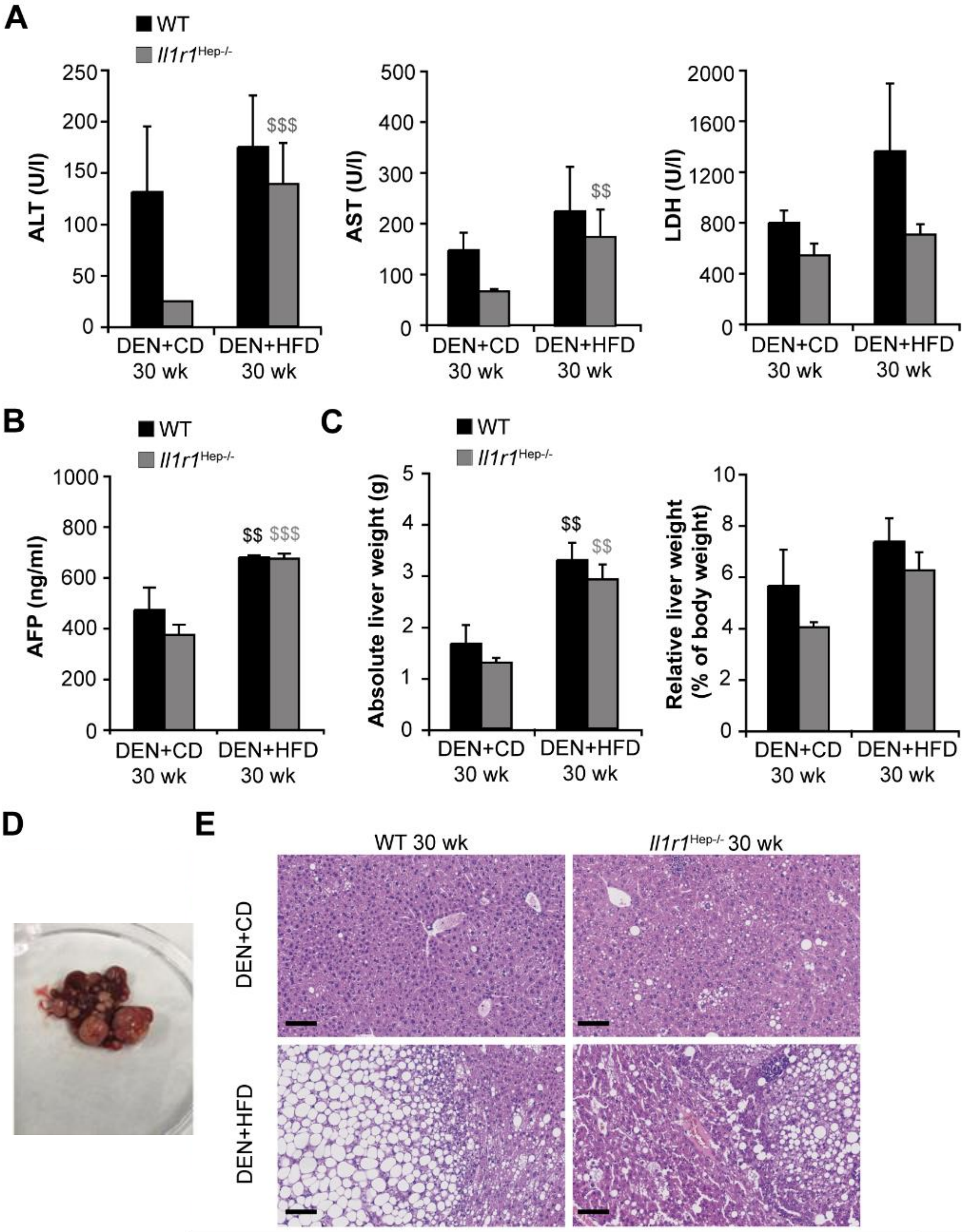

	WT DEN+CD 30 wk	*Il1r1*Hep-/- DEN+CD 30 wk	WT DEN+HFD 30 wk	*Il1r1*Hep-/- DEN+HFD 30 wk
**Steatosis (0-3)**	1.25 ± 0.23	1.14 ± 0.24	2.44 ± 0.15 ($$)	2.83 ± 0.16 ($$)
**Inflammation (0-3)**	0.25 ± 0.15	0.0 ± 0.0	0.56 ± 0.15	0.58 ± 0.14
**Fibrosis (0-3)**	0.25 ± 0.15	0.0 ± 0.0	0.17 ± 0.13	0.0 ± 0.0

Supplementary Figure S5: Effect of prolonged HFD consumption on DEN-initiated liver tumor growth in *Il1r1*$^{Hep-/-}$ and WT mice. Livers from 30-week-old *Il1r1*$^{Hep-/-}$ and WT mice, which had received DEN+HFD or DEN+CD, were evaluated for tumors. (A) Nodules visible at the surface upon macroscopic examination, (B) diameter of the visible nodules, (C) calculated total tumor area, and (D) determination of the ratios of tumor area per whole histological section area (SL: surrounding liver) by digital means. (E) Gene expression analysis of Ki-67 and cyclin D1 in tumor surrounding liver (SL) and individual tumors (T). Expression data were normalized to the housekeeping gene *Gapdh*, which was stably expressed, and calculated as fold change over expression in WT PBS+CD mice, which was considered 1. Data in A-C represent mean ± SEM of n=8 WT DEN+CD, n=16 WT DEN+HFD, n=7 *Il1r1*$^{Hep-/-}$ DEN+CD, n=12 *Il1r1*$^{Hep-/-}$ DEN+HFD mice at 30 weeks of age. Data in D represent mean ± SEM of n=6 WT DEN+CD, n=10 WT DEN+HFD, n=6 *Il1r1*$^{Hep-/-}$ DEN+CD, n=8 *Il1r1*$^{Hep-/-}$ DEN+HFD mice at 30 weeks of age. Data in E represent mean ± SEM of n=7 WT DEN+CD, n=12 WT DEN+HFD, n=6 *Il1r1*$^{Hep-/-}$ DEN+CD, n=9 *Il1r1*$^{Hep-/-}$ DEN+HFD mice at 30 weeks of age. Gene expression in individual tumors was assessed in n=5 mice/genotype. * p<0.05 for WT vs. *Il1r1*$^{Hep-/-}$, and $^{\$}$ <0.05, $^{\$\$}$ p<0.01, $^{\$\$\$}$ p<0.001 for DEN+CD vs. DEN+HFD using Kruskal-Wallis H test followed by pairwise Bonferroni-corrected Mann-Whitney *U* tests (A-D) or one-way ANOVA with post-hoc Tukey tests (E).

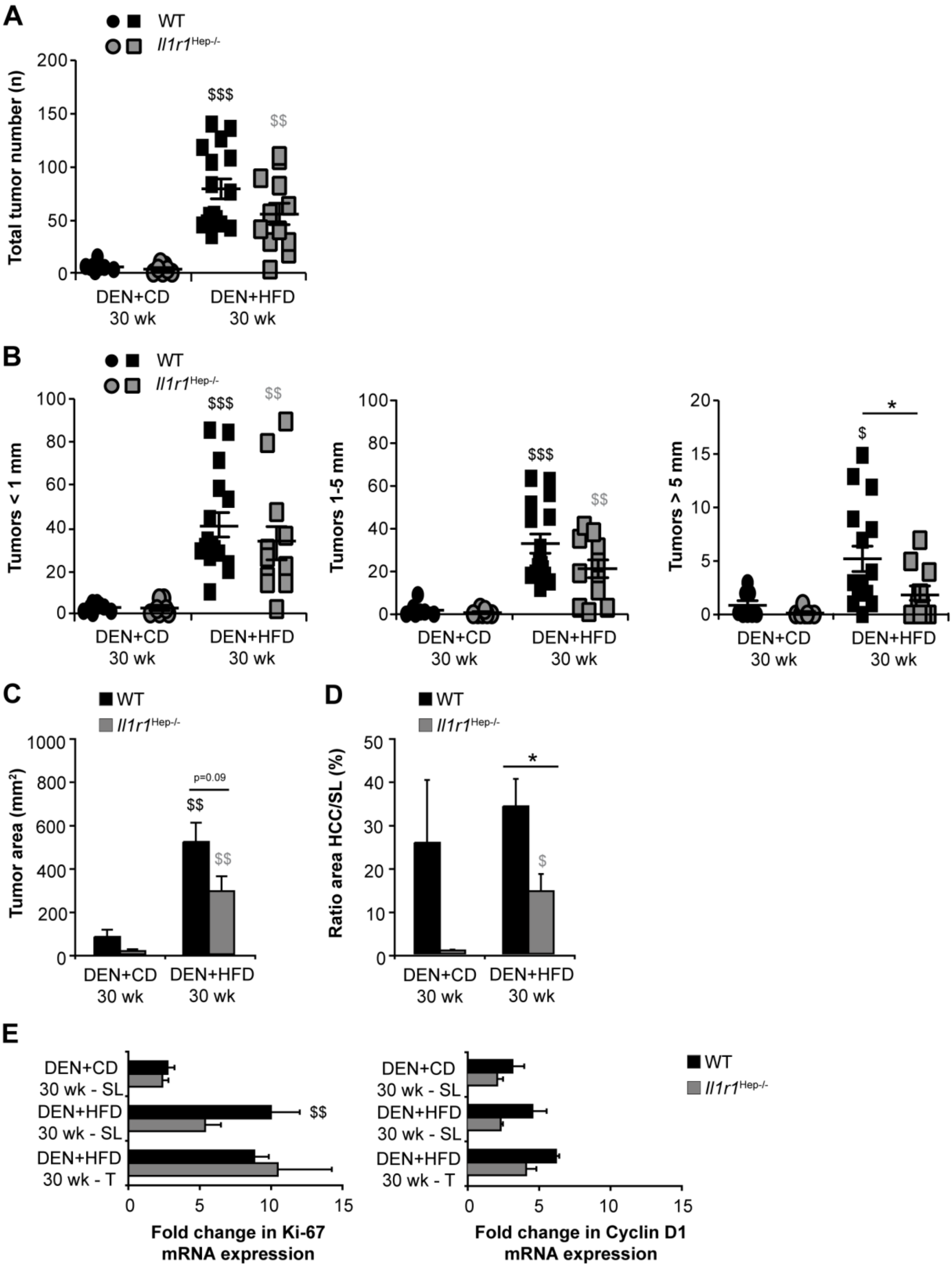
A
WT
Il1r1Hep-/-
Total tumor number (n)
200
150
100
50
0
$$$
$$
DEN+CD 30 wk
DEN+HFD 30 wk
B
WT
Il1r1Hep-/-
Tumors < 1 mm
Tumors 1-5 mm
Tumors > 5 mm
$$$
$$
$
*
DEN+CD 30 wk
DEN+HFD 30 wk
C
WT
Il1r1Hep-/-
Tumor area (mm²)
p=0.09
$$
DEN+CD 30 wk
DEN+HFD 30 wk
D
WT
Il1r1Hep-/-
Ratio area HCC/SL (%)
*
$
DEN+CD 30 wk
DEN+HFD 30 wk
E
DEN+CD 30 wk - SL
DEN+HFD 30 wk - SL
DEN+HFD 30 wk - T
$$
Fold change in Ki-67 mRNA expression
Fold change in Cyclin D1 mRNA expression
WT
Il1r1Hep-/-

Supplementary Table S1: Crude nutrients of experimental diets (both ssniff Spezialdiäten GmbH, Soest, Germany).

**Product No.**		**Surwit Diet (HFD) [HF/sucrose] E15772-34**	**Control Diet (CD) [soybean oil] E15772-04**
Casein	%	23.00	23.00
Corn stach, pre-gelatinized	%	-	27.00
Maltodextrin	%	15.30	15.30
Sucrose	%	17.00	17.00
Pur. Cellulose powder	%	1.80	5.20
DL-Methionine	%	0.10	0.10
L-Cystine	%	0.10	0.10
Mineral & trace element premix	%	5.50	5.50
Vitamin premix	%	1.00	1.00
Sodium phosphate, dibasic	%	0.30	0.30
Dye, red-yellow mix	%	0.10	-
Choline chloride	%	0.20	0.20
Coconut oil, hydrogenated	%	33.30	-
Soybean oil	%	2.30	5.30
ME, Atwater*	MJ/kg	22.80	15.70
Protein	kJ%	15	22
Carbohydrates	kJ%	26	65
Lipids	kJ%	58	13

* physiological fuel value

Supplementary Table S2: Mouse forward and reverse primers used for qRT-PCR.

Protein	Gene	Forward primer	Reverse primer
CPT1	*Cpt1a*	AGT GGC CTC ACA GAC TCC AG	GCC ATG TTG TAC AGC TTC C
Cyclin D1	*Ccnd1*	TGC CAT CCA TGC GGA AA	AGC GGG AAG AAC TCC TCT TC
FXR-α	*Nr1h4*	CCC AGA GAA GAA CCG AGT T	TAG ATG CCA GGA GAA TAC CAG
HO-1	*Hmox1*	CCT CAC TGG CAG GAA ATC ATC	CCT CGT GGA GAC GCT TTA CAT A
IFN-γ	*Ifng*	GCG TCA TTG AAT CAC ACC TG	GAC CTG TGG GTT GTT GAC CT
IL-1α	*Il1a*	CAA ACT GAT GAA GCT CGT CA	TCT CCT TGA GCG CTC ACG AA
IL-1β	*Il1b*	TCT TTG AAG TTG ACG GAC CC	TGA GTG ATA CTG CCT GCC TG
IL-1Ra	*Il1rn*	GGG ATA CTA ACC AGA AGA CC	GAC AGG CAC AGC TTG CCC CC
IL-6	*Il6*	AGT TGC CTT CTT GGG ACT GA	TTC TGC AAG TGC ATC ATC GT
IL-10	*Il10*	ACT GCA CCC ACT TCC CAG T	TGT CCA GCT GGT CCT TTG TT
CCL2	*Ccl2*	CTT CTG GGC CTG CTG TTC A	CCA GCC TAC TCA TTG GGA TCA
CXCL1	*Cxcl1*	ATC CAG AGC TTG AAG GTG TTG	GTC TGT CTT CTT TCT CCG TTA CTT
CXCL2	*Cxcl2*	CTC TCA AGG GCG GTC AAA AAG TT	TCA GAC AGC GAG GCA CAT CAG GTA
Ki-67	*Mki67*	TCT GAT GTT AGG TGT TTG AG	CAC TTT TCT GGT AAC TTC TTG
PPAR-α	*Ppara*	ATG AAG AGG GCT GAG CGT AG	AAA CGC AAC GTA GAG TGC TGT
PPAR-γ	*Pparg*	GAT GGA AGA CCA CTC GCA TT	AAC CAT TGG GTC AGC TCT TG
SREBP1c	*Srebf1*	ATC TCC TAG AGC GAG CGT TG	TAT TTA GCA ACT GCA GAT ATC CAA G
TGF-β	*Tgfb2*	TTC CTG GCG TTA CCT TGG T	CCA CTG CCG GAC AAC T
TNF-α	*Tnf*	GAA GTT CCC AAA TGG CCT CC	GTG AGG GTC TGG GCC ATA GA

Supplementary Table S3: Gene expression analysis of lipid metabolism-associated genes in hepatic tissue from *Il1r1*$^{Hep-/-}$ and WT mice following treatment with DEN+HFD or DEN+CD. qRT-PCR analysis was performed in adjacent non-tumorous liver tissue lysates from 24-week-old *Il1r1*$^{Hep-/-}$ and WT mice exposed to DEN+HFD or DEN+CD and normal liver tissue from PBS+CD controls. Expression data were normalized to the housekeeping gene *Gapdh*, which was stably expressed, and calculated as fold change over expression in WT PBS+CD mice, which was considered 1. (Data represent mean ± SEM of n=3 WT PBS+CD, n=7 WT DEN+CD, n=11-14 WT DEN+HFD, n=3 *Il1r1*$^{Hep-/-}$ PBS+CD, n=7 *Il1r1*$^{Hep-/-}$ DEN+CD, n=11-14 *Il1r1*$^{Hep-/-}$ DEN+HFD mice at 24 weeks of age. $^{\#\#}$ p<0.01, $^{\#\#\#}$ p<0.001 for PBS vs. DEN, and $^{\$\$\$}$ p<0.001 for DEN+CD vs. DEN+HFD using one-way ANOVA with post-hoc Tukey tests. There was no statistical difference between *Il1r1*$^{Hep-/-}$ and WT mice.)

	**WT PBS+CD**	***Il1r1*$^{Hep-/-}$ PBS+CD**	**WT DEN+CD**	***Il1r1*$^{Hep-/-}$ DEN+CD**	**WT DEN+HFD**	***Il1r1*$^{Hep-/-}$ DEN+HFD**
**PPAR-γ**	1.00 ± 0.22	0.77 ± 0.07	0.31 ± 0.04 ($^{\#\#\#}$)	0.38 ± 0.04	0.98 ± 0.08 ($^{\$\$\$}$)	1.11 ± 0.07 ($^{\$\$\$}$)
**SREBP1c**	1.00 ± 0.17	0.76 ± 0.34	0.54 ± 0.08	0.39 ± 0.04	0.81 ± 0.09	0.78 ± 0.07
**PPAR-α**	1.00 ± 0.24	0.88 ± 0.17	0.66 ± 0.13	0.78 ± 0.06	0.68 ± 0.05	0.73 ± 0.05
**CPT1**	1.00 ± 0.36	1.08 ± 0.15	3.15 ± 0.58 ($^{\#\#}$)	3.83 ± 0.21 ($^{\#\#\#}$)	3.60 ± 0.18 ($^{\#\#\#}$)	3.97 ± 0.15 ($^{\#\#\#}$)
**FXR-α**	1.00 ± 0.11	0.98 ± 0.11	1.17 ± 0.13	1.55 ± 0.13	1.45 ± 0.09	1.53 ± 0.09
**HO-1**	1.00 ± 0.15	0.77 ± 0.10	1.66 ± 0.18	2.19 ± 0.30	2.78 ± 0.25	3.88 ± 0.54 ($^{\#\#}$)

Supplementary Table S4: DEN+HFD-induced inflammatory cytokine and chemokine expression in the liver of *Il1r1*$^{Hep-/-}$ and WT mice. qRT-PCR analysis was performed in adjacent non-tumorous liver tissue lysates from 24-week-old *Il1r1*$^{Hep-/-}$ and WT mice post DEN+HFD or DEN+CD treatment and normal liver tissue from PBS+CD controls. Expression data were normalized to the housekeeping gene *Gapdh*, which was stably expressed, and calculated as fold change over expression in WT PBS+CD mice, which was considered 1. (Data represent mean ± SEM of n=3 WT PBS+CD, n=7 WT DEN+CD, n=14 WT DEN+HFD, n=3 *Il1r1*$^{Hep-/-}$ PBS+CD, n=7 *Il1r1*$^{Hep-/-}$ DEN+CD, n=14 *Il1r1*$^{Hep-/-}$ DEN+HFD mice at 24 weeks of age. $^{\#}$ p<0.05, $^{\#\#}$ p<0.01, $^{\#\#\#}$ p<0.001 for PBS vs. DEN, and $^{\$}$ p<0.05, $^{\$\$}$ p<0.01, $^{\$\$\$}$ p<0.001 for DEN+CD vs. DEN+HFD using one-way ANOVA with post-hoc Tukey tests. There was no statistical difference between *Il1r1*$^{Hep-/-}$ and WT mice.)

	**WT PBS+CD**	***Il1r1*$^{Hep-/-}$ PBS+CD**	**WT DEN+CD**	***Il1r1*$^{Hep-/-}$ DEN+CD**	**WT DEN+HFD**	***Il1r1*$^{Hep-/-}$ DEN+HFD**
**IL-1α**	1.00 ± 0.13	1.07 ± 0.03	2.10 ± 0.30	2.25 ± 0.30	3.29 ± 0.20 ($^{\#\#\#}$, $^{\$}$)	3.73 ± 0.29 ($^{\#\#\#}$, $^{\$\$}$)
**IL-1β**	1.00 ± 0.17	0.91 ± 0.29	7.37 ± 1.13	8.86 ± 2.27	15.10 ± 2.04	18.89 ± 3.44 ($^{\#\#}$)
**IL-1Ra**	1.00 ± 0.46	0.71 ± 0.12	8.18 ± 2.33	7.97 ± 1.04	29.08 ± 3.32 ($^{\#\#\#}$, $^{\$\$\$}$)	26.70 ± 3.57 ($^{\#\#\#}$, $^{\$\$}$)
**TNF-α**	1.00 ± 0.27	1.09 ± 0.14	1.14 ± 0.11	1.12 ± 0.19	1.57 ± 0.19	2.12 ± 0.29
**IL-6**	1.00 ± 0.18	1.33 ± 0.25	0.94 ± 0.11	1.35 ± 0.25	1.78 ± 0.23	1.75 ± 0.17
**IL-10**	1.00 ± 0.13	0.94 ± 0.05	1.01 ± 0.15	0.94 ± 0.20	2.28 ± 0.48	3.22 ± 0.47 ($^{\#\#}$)
**IFN-γ**	1.00 ± 0.21	1.59 ± 0.16	4.77 ± 0.98	7.00 ± 1.11 ($^{\#}$)	5.46 ± 0.53 ($^{\#}$)	6.45 ± 0.73 ($^{\#}$)
**TGF-β**	1.00 ± 0.14	1.16 ± 0.12	2.16 ± 0.34	2.44 ± 0.17	2.93 ± 0.23 ($^{\#\#}$)	3.80 ± 0.23 ($^{\#\#\#}$, $^{\$\$}$)
**CCL2**	1.00 ± 0.12	0.80 ± 0.03	2.44 ± 0.40	3.90 ± 0.60	14.41 ± 2.26	17.63 ± 4.04 ($^{\#}$, $^{\$}$)
**CXCL1**	1.00 ± 0.07	0.81 ± 0.17	1.76 ± 0.12	2.16 ± 0.42	3.94 ± 0.58	2.87 ± 0.49
**CXCL2**	1.00 ± 0.06	1.01 ± 0.23	10.32 ± 1.75	10.08 ± 2.14	25.26 ± 3.16 ($^{\#\#\#}$, $^{\$\$}$)	16.84 ± 2.18 ($^{\#}$)
